# Supplementary material for: Understanding the self-assembly dynamics of A/T absent ‘four-way DNA junctions with sticky ends’ at altered physiological conditions through molecular dynamics simulations
Source: PLoS One. 2023 Feb 8;18(2):e0278755. doi: 10.1371/journal.pone.0278755 (PMC9907842; doi:10.1371/journal.pone.0278755)
Supplement: S1 Table — (PDF) [file pone.0278755.s001.pdf]

Supporting information

Understanding the self-assembly dynamics of A/T absent 'four-way DNA junctions with sticky ends' at altered physiological conditions through molecular dynamics simulations

Akanksha Singh[1], Ramesh Kumar Yadav[2], Ali Shati[3], Nitin Kumar Kamboj[4], Hesham Hasssan[5,6], Shiv Bharadwaj[7*], Rashmi Rana[8*], Umesh Yadava[1*]

[1]Departmet of Physics, Deen Dayal Upadhyaya Gorakhpur University, Gorakhpur, 273009 India

[2]Department of Physics, B.R.D. Post Graduate College, Deoria, 274001 India

[3s]Department of Biology, Faculty of Science, King Khaild University, Abha, Saudi Arabia

[4]School of Physical Sciences, DIT University, Dehradun, UK, 248001, India

[5]Department of Pathology, College of Medicine, King Khaild University, Abha, Saudi Arabia

[6]Department of Pathology, Faculty of Medicine, Assiut University, Assiut, Egypt

[7]Department of Biotechnology, Institute of Biotechnology, College of Life and Applied Sciences, Yeungnam University, 280 Daehak-Ro, Gyeongsan, Gyeongbuk, 38541, Republic of Korea

[8]Department of Research, Sir Ganga Ram Hospital, New Delhi, India

*Corresponding authors

Email; SB: shiv@ynu.ac.kr

RR: Rashmi.rana@sgrh.com

UY: u_yadava@yahoo.com

S1 a. Table: Torsion angles parameters of the initial structure of the DNA junction.

Residue	Base	Chi A/S (χ)	Alpha (α)	Beta (β)	Gamma (γ)	Delta (δ)	Epsilon (ε)	Zeta (ζ)	Puckering
1	A1_DC	-147.6anti	---	---	-52.7	127.7	-151.0	-75.8	C1′-endo
2	A1_DG	-107.0 anti	-73.0	177.4	63.1	148.5	-170.0	-113.4	C2′-endo
3	A1_DG	-95.8 anti	-56.3	-171.5	40.7	149.3	-162.8	-129.6	C2′-endo
4	A1_DC	-76.8 anti	-22.5	161.2	15.9	140.4	-160.3	-174.5	C2′-endo
5	A1_DG	-80.3 anti	26.1	115.3	7.2	150.3	-135.3	169.1	C2′-endo
6	A1_DG	-117.5 anti	123.7	-123.7	160.6	124.6	-73.3	-65.3	C1′-exo
7	A1_DC	-144.6 anti	-83.1	-164.7	88.5	138.5	-118.0	-90.2	C3′-exo
8	A1_DC	-62.1 anti	-48.4	167.3	29.6	162.3	-155.4	-149.2	C3′-exo
9	A1_DG	-129.7 anti	-99.8	147.7	73.6	109.1	62.7	91.9	C4′-exo
10	A1_DC	-158.8 anti	175.7	140.3	109.6	102.8	---	---	C4′-endo
11	B1_DC	-75.2 anti	---	---	-116.3	148.7	-11.7	52.0	C2′-endo
12	B1_DG	-95.4 anti	175.9	-110.7	103.1	136.3	172.7	-109.5	C2′-endo
13	B1_DG	-115.7 anti	-70.8	-170.9	57.4	138.3	-170.6	-98.2	C3′-exo
14	B1_DC	-101.4 anti	-71.7	-176.1	53.9	150.2	177.9	-114.1	C3′-exo
15	B1_DG	-112.5 anti	-59.8	-179.4	56.7	125.3	-174.6	-93.9	C2′-endo
16	B1_DG	-91.2 anti	-24.2	171.7	22.7	155.5	-112.4	-178.6	C2′-endo
17	B1_DC	-119.7 anti	-76.3	153.2	51.1	135.0	-142.6	-66.3	C1′-exo
18	B1_DC	-101.2 anti	-39.4	-66.0	-94.8	-177.7	-155.5	-126.1	C3′-exo
19	B1_DG	-65.2 anti	-21.8	164.4	3.8	-174.2	-117.2	153.3	C3′-exo
20	B1_DC	-139.8 anti	101.5	-139.6	162.7	95.5	---	---	C2′-exo
21	C1_DC	-147.3 anti	---	---	50.6	85.8	-172.3	-76.2	C4′-exo
22	C1_DG	-129.4 anti	-112.1	-175.6	94.8	133.1	-166.0	-126.8	C2′-endo
23	C1_DG	-94.6 anti	18.9	174.4	-13.2	165.0	136.5	-84.2	C3′-exo
24	C1_DC	-54.4 anti	-123.7	-99.8	56.5	160.1	-80.4	138.0	C2′-endo
25	C1_DG	-61.3 anti	-69.4	178.6	28.2	154.5	153.0	-86.2	C3′-exo
26	C1_DG	-84.5 anti	-87.5	-170.7	57.9	123.9	-70.6	-96.9	C1′-exo
27	C1_DC	-160.5 anti	-80.0	-164.6	28.3	73.1	99.5	47.3	C3′-endo
28	C1_DC	-105.6 anti	-125.3	-166.6	63.9	140.6	174.6	-89.4	C2′-endo
29	C1_DG	-97.3 anti	-98.5	-169.1	73.9	138.4	-97.0	157.7	C1′-exo
30	C1_DC	-107.6 anti	-41.8	126.2	19.1	141.2	---	---	C2′-endo
31	D1_DC	-50.1 anti	---	---	160.8	159.8	-143.6	165.3	C2′-endo
32	D1_DG	-116.6 anti	-70.3	169.8	37.5	114.6	102.2	49.8	C4′-exo
33	D1_DG	-142.3 anti	-168.1	43.5	-171.9	177.0	-159.4	-68.1	C4′-endo
34	D1_DC	-84.1 anti	-61.7	178.1	29.3	149.5	-153.5	-137.5	C2′-endo
35	D1_DG	-85.6 anti	-50.7	153.8	38.4	144.1	-106.5	176.9	C2′-endo
36	D1_DG	-99.5 anti	-63.3	128.7	48.5	135.8	-167.0	-119.2	C2′-endo
37	D1_DC	-88.7 anti	-19.7	-168.7	-3.5	169.9	-149.2	-132.4	C2′endo
38	D1_DC	-64.2 anti	-36.2	171.9	6.7	167.3	-117.4	170.0	C2′-endo
39	D1_DG	-73.1 anti	-53.9	163.8	33.7	166.0	174.8	-173.7	C3′-exo
40	D1_DC	-149.2 anti	136.0	-97.7	160.5	100.9	---	---	C4′-endo

S1 b. Table: Torsion angle parameters for the final structure after 100 ns MD simulation done at 200K temperature.

**Residue**	**Base**	**Chi A/S (χ)**	**Alpha (α)**	**Beta (β)**	**Gamma (γ)**	**Delta (δ)**	**Epsilon (ε)**	**Zeta (ζ)**	**Puckering**
1	A1_DC	-106.7 anti	---	---	63.4	148.3	-172.5	-88.4	C2'-endo
2	A1_DG	-92.4 anti	-77.1	-176.7	57.8	146.3	173.1	-96.1	C2'-endo
3	A1_DG	-100.1 anti	-55.2	-179.3	53.1	136.8	177.2	-76.9	C3'-exo
4	A1_DC	-152.8 anti	-55.9	169.4	59.1	94.1	-175.9	-86.0	O4'-endo
5	A1_DG	-80.4 anti	-63.0	-161.4	47.7	150.9	-144.5	-174.7	C3'-exo
6	A1_DG	-148.0 anti	98.2	-92.8	-176.8	121.1	-66.8	-48.3	C1'-exo
7	A1_DC	-164.9 anti	-65.5	-154.8	66.1	140.1	-119.9	-69.7	C2'-endo
8	A1_DC	-65.5 anti	-86.6	156.9	55.3	134.2	-126.2	177.8	C1'-exo
9	A1_DG	-128.5 anti	-64.9	131.5	50.0	127.6	57.1	84.1	C2'-endo
10	A1_DC	-74.4 anti	-169.4	-166.5	42.8	140.7	---	---	C1'-exo
11	B1_DC	-87.2 anti	---	---	60.3	126.2	-126.0	178.4	C1'-exo
12	B1_DG	-109.4 anti	-59.4	115.4	64.7	128.8	168.5	-79.4	C2'-endo
13	B1_DG	-89.2 anti	-63.0	-163.1	58.2	145.5	-176.9	-98.7	C3'-exo

14	B1_DC	-117.5 anti	-63.6	165.2	59.2	122.9	173.0	-97.4	C1'-exo
15	B1_DG	-100.1 anti	-68.4	-147.4	50.1	148.6	-153.4	-95.3	C3'-exo
16	B1_DG	-83.3 anti	-73.6	163.9	60.0	135.0	-76.5	141.1	C2'-endo
17	B1_DC	-114.6 anti	-76.6	138.2	41.3	137.6	-131.5	177.4	C1'-exo
18	B1_DC	-134.4 anti	114.9	-91.1	176.9	92.8	-152.4	-84.1	O4'-endo
19	B1_DG	-79.9 anti	-61.4	179.4	40.6	150.4	-133.1	178.9	C2'-endo
20	B1_DC	-158.0 anti	79.3	-112.3	-152.3	134.7	---	---	C2'-endo
21	C1_DC	-107.0 anti	---	---	51.0	148.1	-166.0	-80.4	C2'-endo
22	C1_DG	-73.9 anti	-71.9	-177.4	45.7	148.2	-150.2	168.3	C2'-endo
23	C1_DG	-150.5 anti	103.3	-83.4	173.1	91.1	-175.0	-68.6	C4'-exo
24	C1_DC	-107.0 anti	-72.2	-164.6	67.7	144.2	-134.4	-157.3	C2'-endo
25	C1_DG	-153.5 anti	-69.2	130.0	56.3	77.8	-145.6	-74.6	C4'-exo
26	C1_DG	-102.5 anti	-58.5	172.2	58.9	154.0	-72.4	-68.2	C2'-endo
27	C1_DC	-163.4 anti	-97.8	-166.3	55.6	74.2	51.1	140.2	C2'-exo
28	C1_DC	-87.4 anti	-77.6	108.7	41.6	125.3	-161.9	-138.9	C1'-exo
29	C1_DG	-120.6 anti	-70.3	157.2	64.4	143.0	-151.0	-93.8	C2'-endo
30	C1_DC	-96.0 anti	-63.7	175.0	43.1	138.5	---	---	C1'-exo
31	D1_DC	-173.0 anti	---	---	53.4	120.2	-106.1	-68.9	C1'-exo
32	D1_DG	-99.4 anti	-60.8	157.1	40.9	66.0	89.0	69.6	C3'-endo
33	D1_DG	-94.7 anti	170.3	78.0	157.7	167.9	-164.1	-104.1	C3'-exo
34	D1_DC	-119.2 anti	-65.2	164.5	61.4	125.5	-144.8	-96.4	C1'-exo
35	D1_DG	-86.4 anti	-53.5	160.8	36.2	138.5	-121.1	172.7	C1'-exo
36	D1_DG	-114.2 anti	-64.6	134.4	57.5	143.0	-170.0	-101.0	C2'-endo
37	D1_DC	-120.7 anti	-79.9	-173.5	45.3	114.3	-159.6	-107.0	O4'-endo
38	D1_DC	-130.4 anti	-56.1	159.1	62.1	127.3	-156.0	-86.8	C1'-exo
39	D1_DG	-83.4 anti	-76.4	175.4	41.2	136.2	-150.6	166.2	C1'-exo
40	D1_DC	-151.2 anti	83.9	-82.4	-163.4	127.5	---	---	C1'-exo

S1 c. Table: Torsion angle parameters for the final structure after 100 ns MD simulation done at 300K temperature.

Residue	Base	Chi A/S (χ)	Alpha (α)	Beta (β)	Gamma (γ)	Delta (δ)	Epsilon (ε)	Zeta (ζ)	Puckering
1	A1_DC	58.8 syn	- - -	- - -	47.3	147.6	-88.8	-74.4	C2'-endo
2	A1_DG	-79.6 anti	-75.3	76.1	172.5	132.1	-118.0	151.3	C1'-exo
3	A1_DG	-95.5 anti	-62.2	136.5	48.3	157.1	-147.7	-74.0	C2'-endo
4	A1_DC	-167.3 anti	-96.0	59.0	-173.7	134.1	-137.1	-62.0	C2'-endo
5	A1_DG	-107.2 anti	-86.2	161.3	43.5	109.1	-149.5	-75.0	C1'-exo
6	A1_DG	-149.6 anti	-78.9	42.1	-173.7	155.0	-109.4	-55.7	C3'-exo
7	A1_DC	169.1 anti	-53.9	-131.5	47.0	135.1	-160.7	-76.7	C1'-exo
8	A1_DC	-80.1 anti	-170.4	-113.9	65.2	144.7	-143.9	-76.5	C2'-endo
9	A1_DG	-173.8 anti	-102.3	74.6	176.5	73.1	-131.2	-76.9	C4'-exo
10	A1_DC	-95.6 anti	-66.8	164.9	63.3	135.2	---	---	C2'-endo
11	B1_DC	56.3 syn	---	---	63.7	121.3	-146.3	-115.8	C1'-exo
12	B1_DG	156.8	-77.6	76.9	172.8	159.3	-108.9	149.9	C3'-exo
13	B1_DG	-100.3 anti	-63.8	124.2	68.8	135.7	175.8	-86.8	C3'-exo
14	B1_DC	-150.0 anti	-67.4	-176.0	38.5	87.3	173.2	-74.3	C3'-endo
15	B1_DG	-88.7 anti	-68.8	-169.6	75.8	161.5	-139.1	-76.7	C3'-exo
16	B1_DG	-177.8 anti	-90.4	68.9	179.0	86.9	-147.2	-80.8	O4'-endo
17	B1_DC	-141.1 anti	-61.0	167.3	42.9	112.0	-109.5	-67.2	O4'-endo
18	B1_DC	-145.0 anti	-83.1	54.5	168.5	132.5	-157.6	-73.2	C1'-exo
19	B1_DG	-83.3 anti	-116.1	-159.2	58.8	132.9	174.3	-84.4	C2'-endo
20	B1_DC	-135.5 anti	-56.4	169.5	60.1	106.8	---	---	O4'-endo
21	C1_DC	47.5 syn	---	---	58.3	102.0	-149.2	-73.8	O4'-endo
22	C1_DG	-74.4 anti	-59.3	162.3	57.3	155.5	-104.0	164.6	C2'-endo
23	C1_DG	-90.0 anti	-73.7	146.0	45.5	143.7	-149.4	-70.2	C2'-endo
24	C1_DC	-169.4 anti	-96.7	57.5	-176.2	144.1	-161.8	-71.7	C1'-exo
25	C1_DG	-82.4 anti	-130.3	-152.8	57.2	143.7	-117.6	179.1	C2'-endo
26	C1_DG	-98.4 anti	-74.5	145.6	47.6	148.5	-114.9	-169.9	C3'-exo
27	C1_DC	-169.0 anti	71.0	150.6	55.0	152.7	-135.9	-63.3	C2'-endo
28	C1_DC	-122.7 anti	-67.3	164.1	23.7	109.0	179.8	-84.9	O4'-endo
29	C1_DG	-89.1 anti	-55.4	-177.0	32.0	147.8	-143.5	-146.6	C2'-endo
30	C1_DC	-70.1 anti	-60.9	162.7	45.3	144.4	---	---	C2'-endo
31	D1_DC	-107.1 anti	---	---	61.3	148.2	-177.3	-86.4	C2'-endo

32	D1_DG	-86.0 anti	-73.6	-174.0	48.1	156.2	-108.6	164.4	C2'-endo
33	D1_DG	-94.4 anti	-63.8	129.1	46.6	137.0	-171.3	-103.0	C2'-endo
34	D1_DC	-95.5 anti	-58.1	-174.2	38.7	145.0	-145.6	174.9	C1'-exo
35	D1_DG	-137.2 anti	-51.5	125.7	56.5	135.7	-152.2	-83.2	C2'-endo
36	D1_DG	-156.5 anti	-67.3	173.3	52.1	100.9	-172.6	-68.2	C1'-exo
37	D1_DC	-112.9 anti	-82.4	-166.3	56.8	119.5	-152.7	-92.0	C1'-exo
38	D1_DC	-108.0 anti	-65.5	162.4	51.7	102.2	165.8	-79.6	O4'-endo
39	D1_DG	-154.3 anti	-49.3	166.0	55.2	82.0	-165.8	-84.2	C4'-exo
40	D1_DC	-110.6 anti	-69.3	-158.0	58.0	142.5	---	---	C2'-endo

S1 d. Table: Torsion angle parameters for the final structure after 100 ns MD simulation done at 310K temperature.

Residue	Base	Chi A/S (χ)	Alpha (α)	Beta (β)	Gamma (γ)	Delta (δ)	Epsilon (ε)	Zeta (ζ)	Puckering
1	A1_DC	-63.1 anti	---	---	60.2	127.8	-153.9	-77.7	C1'-exo
2	A1_DG	-153.1 anti	-99.6	70.9	-177.9	94.3	-173.5	-93.9	O4'-endo
3	A1_DG	-88.1 anti	-63.9	-163.3	66.9	139.3	-169.9	-101.8	C3'-exo
4	A1_DC	-121.0 anti	-45.0	148.1	48.4	94.2	-168.9	-98.1	O4'-endo
5	A1_DG	-88.7 anti	-68.7	175.5	69.5	147.6	171.0	-100.1	C3'-exo
6	A1_DG	-78.2 anti	-66.8	-143.5	39.2	165.3	-95.9	-83.5	C3'-exo
7	A1_DC	-115.7 anti	-120.1	-104.3	58.7	128.1	-155.9	-82.4	C2'-endo
8	A1_DC	-151.0 anti	-73.4	176.5	44.3	116.2	177.7	-97.4	C1'-exo
9	A1_DG	-114.3 anti	-47.5	-161.1	50.1	140.8	-177.6	-91.6	C2'-endo
10	A1_DC	-113.0 anti	-78.7	-161.4	37.5	97.9	---	---	C4'-exo
11	B1_DC	-104.8 anti	---	---	44.6	121.5	173.3	-83.1	C1'-exo
12	B1_DG	-101.0 anti	-73.2	-175.7	57.9	153.0	-148.2	-156.2	C2'-endo
13	B1_DG	-113.2 anti	-47.1	145.0	57.4	132.7	-159.1	-69.3	C3'-exo
14	B1_DC	-98.6 anti	-72.9	172.7	42.0	140.0	-117.3	174.7	C1'-exo
15	B1_DG	-114.2 anti	-66.4	141.0	44.9	135.8	163.4	-83.2	C2'-endo
16	B1_DG	-120.7 anti	-73.2	-152.8	46.4	137.7	179.2	-98.7	C2'-endo
17	B1_DC	-107.3 anti	-56.2	-173.0	51.0	137.9	-169.4	-72.7	C2'-endo
18	B1_DC	-139.1 anti	-71.5	176.5	45.7	84.5	-158.8	-93.9	O4'-endo
19	B1_DG	-122.6 anti	-64.1	168.0	56.2	115.8	-160.4	-96.5	C1'-exo
20	B1_DC	-108.2 anti	-68.0	174.7	59.0	119.1	---	---	C2'-endo
21	C1_DC	-155.4 anti	---	---	71.8	156.6	-123.9	-167.4	C2'-endo
22	C1_DG	-138.5 anti	-84.8	57.4	164.6	93.9	-173.9	-90.1	O4'-endo
23	C1_DG	-75.2 anti	-56.6	175.1	69.7	160.5	-172.8	-101.0	C3'-exo
24	C1_DC	-106.2 anti	-76.0	176.9	63.2	132.5	-128.8	-146.5	C1'-exo
25	C1_DG	-157.7 anti	-100.8	148.8	54.7	101.4	172.3	-95.4	C3'-endo
26	C1_DG	-143.2 anti	153.4	-145.0	-174.0	171.8	-83.2	-47.4	C2'-endo
27	C1_DC	-160.5 anti	-91.8	-138.4	67.5	128.8	-143.2	-85.1	C1'-exo
28	C1_DC	-152.9 anti	-59.5	166.0	47.9	92.8	-160.9	-81.3	O4'-endo
29	C1_DG	-86.1 anti	-69.3	-175.3	62.8	163.7	-114.6	157.9	C3'-exo
30	C1_DC	-109.3 anti	-55.7	138.9	35.3	145.4	---	---	C2'-endo
31	D1_DC	-116.9 anti	---	---	57.4	143.8	-116.9	-173.1	C1'-exo
32	D1_DG	-109.5 anti	-82.4	155.2	54.2	148.2	-141.6	-87.4	C3'-exo
33	D1_DG	-60.2 anti	-62.8	162.3	42.6	149.5	-142.7	-169.6	C2'-endo
34	D1_DC	-153.9 anti	-65.5	120.0	66.6	71.5	-147.8	-78.5	C3'-endo
35	D1_DG	-125.4 anti	-52.8	177.4	63.4	149.9	-153.5	-96.9	C3'-exo
36	D1_DG	-160.2 anti	-70.0	177.7	52.3	83.1	-167.8	-65.1	C4'-exo
37	D1_DC	-138.0 anti	-80.4	-170.5	55.9	107.9	-179.2	-92.2	O4'-endo
38	D1_DC	-127.8 anti	-54.3	169.5	64.1	135.6	-128.9	-100.0	C1'-exo
39	D1_DG	-92.1 anti	-67.9	143.4	55.2	137.8	-112.4	175.8	C1'-exo
40	D1_DC	-117.2 anti	-62.9	130.8	42.4	138.2	---	---	C2'-endo
