# Supplementary material for: Understanding the self-assembly dynamics of A/T absent ‘four-way DNA junctions with sticky ends’ at altered physiological conditions through molecular dynamics simulations
Source: PLoS One. 2023 Feb 8;18(2):e0278755. doi: 10.1371/journal.pone.0278755 (PMC9907842; doi:10.1371/journal.pone.0278755)
Supplement: S2 Table — (PDF) [file pone.0278755.s002.pdf]

Supporting information

Understanding the self-assembly dynamics of A/T absent 'four-way DNA junctions with sticky ends' at altered physiological conditions through molecular dynamics simulations

Akanksha Singh[1], Ramesh Kumar Yadav[2], Ali Shati[3], Nitin Kumar Kamboj[4], Hesham Hasssan[5,6], Shiv Bharadwaj[7*], Rashmi Rana[8*], Umesh Yadava[1*]

[1]Departmet of Physics, Deen Dayal Upadhyaya Gorakhpur University, Gorakhpur, 273009 India

[2]Department of Physics, B.R.D. Post Graduate College, Deoria, 274001 India

[3s]Department of Biology, Faculty of Science, King Khaild University, Abha, Saudi Arabia

[4]School of Physical Sciences, DIT University, Dehradun, UK, 248001, India

[5]Department of Pathology, College of Medicine, King Khaild University, Abha, Saudi Arabia

[6]Department of Pathology, Faculty of Medicine, Assiut University, Assiut, Egypt

[7]Department of Biotechnology, Institute of Biotechnology, College of Life and Applied Sciences, Yeungnam University, 280 Daehak-Ro, Gyeongsan, Gyeongbuk, 38541, Republic of Korea

[8]Department of Research, Sir Ganga Ram Hospital, New Delhi, India

*Corresponding authors

Email; SB: shiv@ynu.ac.kr

RR: Rashmi.rana@sgrh.com

UY: u_yadava@yahoo.com

S2 a. Table: Helix axis parameters(intra) of the initial structure of the DNA Junction.

Base pair	Shear	Stretch	Stagger	Buckle	Propeller	Opening	Shift	Slide	Rise	Tilt	Roll	Twist
G-C	-1.321	-0.283	-0.385	3.911	-8.405	11.791	0.000	0.000	0.000	0.000	0.000	0.000
C-G	0.323	-0.383	-0.881	7.719	-5.377	-3.585	-0.205	0.940	3.049	4.473	-0.132	47.092
G-C	-0.253	-0.548	0.068	3.439	-7.161	-3.071	-0.267	1.525	3.515	-2.587	9.253	33.051
G-C	1.009	-0.329	0.567	6.267	-12.032	-1.807	-0.091	1.164	3.316	-3.394	-1.164	44.331
C-G	-0.270	-0.062	0.833	-8.052	-16.319	2.145	1.132	-0.191	3.659	4.438	-2.386	29.754
C-G	-0.273	-0.030	0.619	1.843	-8.952	2.023	-0.578	-0.242	3.174	1.673	9.962	30.180
G-C	0.383	0.291	0.120	2.896	-14.022	5.176	0.572	0.372	3.291	4.242	6.270	34.190
C-G	-0.928	-0.486	0.479	-3.581	-5.290	-3.314	-0.600	0.572	3.505	-6.784	-0.436	38.653
G-C	-0.604	0.145	0.379	5.574	-11.851	-2.031	18.640	-5.230	-4.026	-131.74	31.539	-76.666
C-G	-0.648	0.216	0.271	0.126	-10.74	4.998	0.319	0.457	3.366	1.979	-0.570	32.564
G-C	0.993	0.230	-0.333	-1.660	-7.103	-6.325	-0.623	2.860	3.363	2.625	-0.190	57.238
G-C	0.722	0.126	-0.131	0.502	-11.417	-12.544	0.154	0.316	3.342	-3.934	10.869	21.557
C-G	0.664	-0.363	0.026	2.400	-17.079	-0.037	1.175	-0.445	3.231	2.350	1.095	29.644
C-G	0.190	0.083	-0.019	1.113	-11.424	8.385	0.278	2.420	3.397	4.192	3.895	40.461
G-C	-0.921	0.212	0.215	-4.466	-11.209	3.299	-0.641	1.539	3.354	-5.000	3.846	35.302
C-G	-0.070	-0.307	0.956	-9.212	-4.321	-6.305	-0.693	0.431	3.738	-6.271	-2.336	42.121

S2 b. Table: Helix axis parameters for the final structure after 100 ns MD simulation done at 200K temperature.

Base pair	Shear	Stretch	Stagger	Buckle	Propeller	Opening	Shift	Slide	Rise	Tilt	Roll	Twist
G-C	-0.459	-0.166	-0.052	-7.690	-9.239	-0.921	0.000	0.000	0.000	0.000	0.000	0.000
C-G	0.230	-0.293	0.823	-13.862	-6.233	-1.611	0.377	-0.434	3.556	-5.970	-4.743	31.940
G-C	-0.217	-0.167	-0.262	-3.899	-21.180	4.978	-0.194	0.735	3.259	8.803	2.818	35.796
G-C	0.019	-0.229	0.422	-1.085	-20.199	-1.919	-0.094	1.859	3.292	-3.894	-6.629	41.489
C-G	-0.188	-0.247	0.152	-2.212	-0.523	-1.817	0.640	0.423	3.454	2.529	-7.543	38.651
C-G	-0.093	-0.074	0.023	15.328	2.997	-2.220	-0.111	1.086	3.274	3.874	11.382	24.619
G-C	0.182	-0.123	0.297	10.629	-13.398	-2.845	0.051	0.275	3.438	-1.498	7.516	42.805
C-G	0.473	-0.137	-0.254	11.648	-17.435	5.674	0.691	-0.302	3.326	7.693	2.984	32.579
G-C	-0.057	-0.110	-0.446	-27.778	-13.431	-3.828	16.631	-7.736	-2.279	-129.851	38.753	-91.485
C-G	0.299	-0.107	0.155	-8.560	11.271	-0.980	-0.536	-0.173	3.082	-5.776	-3.657	30.228
G-C	-0.042	-0.136	0.109	2.478	-8.616	3.370	-1.136	0.442	3.080	-2.511	-8.531	32.696
G-C	0.201	-0.195	0.440	8.491	0.567	-4.716	0.175	0.270	3.344	0.336	16.519	31.586
C-G	0.306	-0.306	0.362	-3.308	-20.517	2.231	1.066	-1.121	3.659	3.918	1.623	29.006
C-G	0.082	-0.339	-0.865	14.469	-11.225	-1.365	0.239	1.555	2.937	11.431	6.421	40.651
G-C	0.341	-0.268	-0.155	-10.143	-13.913	0.083	-1.010	1.158	4.159	-12.529	5.015	35.737
C-G	0.022	-0.143	0.345	-11.362	-7.905	-2.490	-0.124	-0.225	3.334	-1.893	1.372	34.091

S2 c. Table: Helix axis parameters for the final structure after 100 ns MD simulation done at 300K temperature.

Base pair	Shear	Stretch	Stagger	Buckle	Propeller	Opening	Shift	Slide	Rise	Tilt	Roll	Twist
G-C	-1.819	6.801	0.854	52.383	21.292	163.493	0.000	0.000	0.000	0.000	0.000	0.000
G-C	-0.444	-0.214	0.389	6.766	-1.439	-0.880	-0.033	3.536	2.603	24.516	-19.578	113.901
C-G	-0.057	0.007	0.559	-7.736	-14.734	-1.965	-0.378	-1.002	3.889	-0.039	-2.114	23.710
G-C	-0.139	-0.198	-0.520	-14.134	-32.190	-3.017	0.298	0.156	3.572	6.564	11.319	38.385
G-C	-0.878	-0.425	-0.193	-8.565	-32.917	1.374	0.227	0.605	3.370	-4.442	2.247	25.342
C-G	-0.022	-0.306	0.283	-19.075	-20.808	-8.873	0.504	-1.362	3.668	0.183	-0.671	40.633
C-G	-0.125	0.050	-0.310	-11.577	-0.464	2.501	-0.830	-0.566	3.307	2.144	1.027	27.212
G-C	-0.554	0.191	0.132	10.312	-6.719	10.165	0.524	0.173	3.015	-9.857	6.289	22.610
C-G	-0.224	-0.058	-0.845	25.855	7.373	0.953	-0.697	0.172	3.322	9.155	-1.039	33.053
G-C	-0.154	-0.156	0.067	-4.380	12.441	-2.826	13.004	13.324	3.889	140.429	69.460	-5.983

C-G	0.091	-0.360	0.634	1.518	-13.342	-6.217	0.971	-0.893	3.571	-4.857	-1.529	27.748
G-C	-0.122	0.044	-0.310	-1.995	0.658	5.268	-0.050	-1.071	3.472	7.968	3.511	28.384
G-C	0.415	-0.170	-0.073	13.843	-9.769	0.258	-1.100	1.219	3.107	-1.379	3.641	38.303
C-G	0.939	-0.245	0.332	5.625	-21.972	5.776	0.056	-0.667	3.486	-3.215	9.000	43.127
C-G	0.111	-0.101	-0.107	-11.502	-12.453	-2.152	-0.795	-2.074	3.796	10.046	7.041	29.903
G-C	0.018	-0.218	-0.252	-7.316	-1.232	-1.780	1.761	-0.130	3.440	5.280	-4.456	24.088
C-G	-0.206	-0.210	-0.423	11.644	23.108	-3.286	-0.305	-0.486	3.425	0.555	11.973	24.212

S2 d. Table: Helix axis parameters for the final structure after 100 ns MD simulation done at 310K temperature.

Base Pair	Shear	Stretch	Stagger	Buckle	Propeller	Opening	Shift	Slide	Rise	Tilt	Roll	Twist
G-C	-0.498	-0.236	-0.197	-12.108	1.904	-0.697	0.000	0.000	0.000	0.000	0.000	0.000
C-G	0.117	-0.029	0.088	9.675	-1.902	3.058	0.568	0.298	2.887	-3.926	4.481	32.843
G-C	-0.207	-0.183	0.616	14.783	-13.293	-0.915	-0.167	0.804	3.691	1.374	8.404	30.167
G-C	-0.274	0.003	-0.400	5.393	-6.685	2.172	-0.651	-0.753	3.563	5.937	10.163	29.324
C-G	-0.225	0.051	0.082	5.997	-10.056	-0.236	0.983	-0.048	3.384	-0.024	3.557	30.856
C-G	0.483	-0.345	0.619	-8.823	-14.438	-0.688	0.373	-0.882	3.739	-0.345	-0.670	44.072
G-C	0.458	-0.175	0.079	1.700	6.920	-2.162	0.341	0.749	3.146	4.614	-2.174	38.613
C-G	-0.221	-0.210	0.020	6.363	-19.353	-1.795	-1.011	0.176	3.447	-1.028	-10.439	31.042
G-C	-0.244	-0.262	-0.401	1.615	-18.844	3.626	12.719	-1.346	-15.574	-15.788	11.586	12.560
C-G	0.188	-0.303	0.570	-9.459	-11.894	-2.227	0.729	0.446	3.739	-5.149	-3.733	36.703
G-C	0.298	-0.137	0.560	-2.121	-12.631	-3.863	-1.077	0.871	3.140	-2.111	3.159	37.980
G-C	-0.403	-0.364	0.173	4.041	-4.324	-0.293	0.496	-1.218	3.448	3.478	-2.116	28.711
C-G	-0.114	-0.062	-1.015	25.850	-5.884	2.364	0.161	-0.565	3.013	12.140	7.330	36.200
C-G	0.190	-0.210	-0.402	-6.606	8.053	-3.986	-0.382	-2.043	4.374	-8.481	10.570	30.387
G-C	-0.372	-0.108	-0.298	-13.576	-19.790	0.977	-0.476	-1.188	4.043	3.890	-2.377	33.377
C-G	0.496	-0.181	-0.029	-17.608	-4.045	-0.054	-0.540	0.175	3.529	-3.187	4.474	44.826
