# Supplementary material for: Understanding the self-assembly dynamics of A/T absent ‘four-way DNA junctions with sticky ends’ at altered physiological conditions through molecular dynamics simulations
Source: PLoS One. 2023 Feb 8;18(2):e0278755. doi: 10.1371/journal.pone.0278755 (PMC9907842; doi:10.1371/journal.pone.0278755)
Supplement: S3 Table — (PDF) [file pone.0278755.s003.pdf]

Supporting information

Understanding the self-assembly dynamics of A/T absent 'four-way DNA junctions with sticky ends' at altered physiological conditions through molecular dynamics simulations

Akanksha Singh[1], Ramesh Kumar Yadav[2], Ali Shati[3], Nitin Kumar Kamboj[4], Hesham Hasssan[5,6], Shiv Bharadwaj[7*], Rashmi Rana[8*], Umesh Yadava[1*]

[1]Departmet of Physics, Deen Dayal Upadhyaya Gorakhpur University, Gorakhpur, 273009 India

[2]Department of Physics, B.R.D. Post Graduate College, Deoria, 274001 India

[3s]Department of Biology, Faculty of Science, King Khaild University, Abha, Saudi Arabia

[4]School of Physical Sciences, DIT University, Dehradun, UK, 248001, India

[5]Department of Pathology, College of Medicine, King Khaild University, Abha, Saudi Arabia

[6]Department of Pathology, Faculty of Medicine, Assiut University, Assiut, Egypt

[7]Department of Biotechnology, Institute of Biotechnology, College of Life and Applied Sciences, Yeungnam University, 280 Daehak-Ro, Gyeongsan, Gyeongbuk, 38541, Republic of Korea

[8]Department of Research, Sir Ganga Ram Hospital, New Delhi, India

*Corresponding authors

Email; SB: shiv@ynu.ac.kr

RR: Rashmi.rana@sgrh.com

UY: u_yadava@yahoo.com

S3 a. Table: Torsion angles parameters of the MD simulated structure at 300K and pH =5 at 100.0 ns

Strand I

base	alpha	beta	gamma	delta	epsilon	zeta	chi
1 G	-58.3	122.0	48.9	148.2	-137.6	-170.6	-85.2
2 C	-47.0	118.0	47.5	97.8	-165.6	-91.8	-135.5
3 G	-65.5	174.8	54.5	142.9	-168.4	-107.9	-139.1
4 G	-68.2	-137.8	43.1	158.9	-88.0	-73.6	-77.4
5 C	-96.7	-130.8	58.5	148.7	-177.1	-74.8	-104.2
6 C	-74.7	-177.3	54.4	102.0	-154.7	-104.3	-160.6
7 G	-72.9	175.9	59.2	134.9	-144.7	-111.4	-126.3
8 C	-67.7	166.3	54.4	130.8	---	---	-113.7
9 G	-66.1	131.3	54.9	117.4	174.1	-92.8	-112.1
10 C	-66.6	169.2	76.1	136.5	-155.0	-67.0	-98.9
11 G	-97.8	53.2	-176.2	136.8	-135.0	-68.1	-165.5
12 G	-107.8	172.3	70.4	141.6	-85.1	-76.3	-94.0
13 C	-63.9	-152.5	58.8	154.1	-134.1	-76.0	-135.0
14 C	-88.8	167.0	52.2	136.1	-105.0	166.5	-62.5
15 G	-66.0	129.8	40.3	152.3	-171.1	-98.5	-94.4
16 C	-72.6	-175.5	57.8	125.3	---	---	-123.3

Strand II

base	alpha	beta	gamma	delta	epsilon	zeta	chi
1 C	-70.2	167.0	63.7	131.5	---	---	-114.0
2 G	-77.3	173.9	60.3	88.4	-157.1	-62.1	-141.1
3 C	-65.8	114.2	64.0	78.0	-144.9	-60.1	-170.9
4 C	-75.7	171.0	44.2	129.4	-137.7	-166.7	-85.9
5 G	-71.5	-132.7	53.2	150.4	-159.2	-91.2	-116.7
6 G	-57.3	162.5	58.4	140.1	168.8	-86.1	-125.9
7 C	-65.9	115.4	55.9	90.8	-153.7	-80.3	-158.0
8 G	-71.7	168.2	45.4	135.8	-133.8	-178.2	-80.5
9 C	-72.0	-177.5	53.8	135.8	---	---	-99.9
10 G	-62.0	-149.6	47.3	139.5	-166.8	-89.7	-127.6
11 C	-83.8	159.8	46.7	102.7	139.4	-70.2	-94.6
12 C	-97.0	70.6	177.7	116.4	-125.4	-60.4	166.7
13 G	-70.9	160.7	45.4	159.0	-154.1	-67.3	-91.6
14 G	-81.6	169.2	55.9	146.2	-131.2	-165.0	-77.6
15 C	-43.7	177.1	62.5	146.2	-139.1	-94.8	-116.7
16 G	-55.4	130.6	36.0	98.9	159.6	-87.0	-118.3

S3 b. Table: Torsion angles parameters of the MD simulated structure at 300K and pH =6 at 100.0 ns

Strand I

base	alpha	beta	gamma	delta	epsilon	zeta	chi
1 G	-106.0	72.5	172.9	131.8	-147.6	-68.7	167.3
2 C	-95.5	-159.6	42.8	140.9	-161.3	-124.4	-122.6
3 G	-62.9	-161.1	46.2	150.6	151.6	-91.9	-73.7
4 G	-63.2	-136.8	47.5	164.8	-83.3	-94.2	-62.3
5 C	-63.2	-125.2	70.1	119.5	-94.4	-102.5	-166.9
6 C	-56.5	131.9	49.4	126.1	-143.7	-156.5	-89.4
7 G	82.3	-128.2	-158.2	87.8	-137.5	-77.4	-166.2
8 C	-56.6	167.2	67.2	140.8	---	---	-92.2
9 G	-87.5	62.9	160.4	152.3	-170.3	-89.2	-118.2
10 C	-67.6	161.0	71.0	134.1	-130.6	-86.3	-147.7
11 G	-80.5	160.2	39.5	137.4	-97.8	153.9	-102.0
12 G	-74.7	132.4	51.1	119.1	-84.4	-61.8	-142.7
13 C	-75.3	-117.1	58.2	145.4	-126.6	-73.7	-140.1
14 C	-81.2	149.8	43.4	131.2	-131.6	-176.4	-63.7
15 G	-65.1	127.8	57.4	155.4	-153.1	-125.1	-121.8
16 C	-70.1	155.8	63.3	137.9	---	---	-116.1

Strand II

base	alpha	beta	gamma	delta	epsilon	zeta	chi
1 C	-55.6	177.9	44.2	93.7	---	---	-143.3
2 G	-76.6	142.9	56.7	154.6	-170.4	-88.0	-104.5
3 C	-73.6	172.5	54.9	146.9	-132.4	-177.0	-84.8
4 C	-68.7	149.9	66.0	95.9	-148.7	-70.2	-131.2
5 G	-46.2	169.7	47.9	140.3	-161.2	-112.5	-86.1
6 G	-57.3	-151.0	39.8	135.9	-176.9	-94.6	-96.2
7 C	-58.4	129.7	41.2	128.9	172.8	-88.5	-129.7
8 G	-118.3	79.8	153.8	146.8	-118.0	171.3	-79.2
9 C	-64.6	-144.7	47.4	147.9	---	---	-102.5
10 G	-46.9	127.9	51.2	135.8	167.5	-98.7	-112.2
11 C	-67.5	-179.1	55.1	140.0	-145.1	-167.0	-62.0
12 C	-75.6	-149.2	46.4	150.3	-175.6	-95.6	-88.4
13 G	-39.5	125.4	57.2	141.9	-169.6	-104.4	-127.4
14 G	-51.5	174.2	40.5	156.1	-152.6	-161.5	-99.3
15 C	-59.4	157.2	61.1	87.8	-153.6	-98.9	-154.0
16 G	96.9	-152.9	-173.0	155.5	-167.9	-99.2	-92.9

S3 c. Table: Torsion angles parameters of the MD simulated structure at 300K and pH = 7 at 100.0 ns

Strand I

base	alpha	beta	gamma	delta	epsilon	zeta	chi
1 G	-75.4	76.1	172.5	132.0	-118.0	151.4	-79.6
2 G	-62.2	136.5	48.3	157.1	-147.7	-74.0	-95.5
3 C	-96.1	59.0	-173.8	134.1	-137.1	-62.0	-167.2
4 G	-86.2	161.3	43.4	109.1	-149.6	-75.0	-107.2
5 G	-79.0	42.2	-173.7	154.9	-109.4	-55.7	-149.7
6 C	71.1	150.6	55.0	152.7	-135.9	-63.3	-169.0
7 C	-67.4	164.1	23.8	108.9	179.8	-84.9	-122.8
8 G	-55.4	-177.0	32.1	147.8	-143.5	-146.6	-88.9
9 C	-60.9	162.7	45.3	144.4	---	---	-70.0
10 G	-73.7	146.1	45.5	143.7	-149.4	-70.3	-90.1
11 C	-96.7	57.5	-176.2	144.0	-161.9	-71.7	-169.3
12 G	-130.3	-152.8	57.1	143.7	-117.6	179.1	-82.4
13 G	-74.5	145.7	47.6	148.6	-115.0	-169.8	-98.4
14 C	-53.9	-131.5	47.1	135.0	-160.7	-76.7	169.1
15 C	-170.3	-114.0	65.2	144.6	-143.9	-76.5	-80.1
16 G	-102.3	74.5	176.5	73.2	-131.2	-76.9	-173.8
17 C	-66.7	164.9	63.2	135.2	---	---	-95.6

Strand II

base	alpha	beta	gamma	delta	epsilon	zeta	chi
1 C	---	---	47.3	147.7	-88.8	-74.5	58.9
2 C	-56.3	169.4	60.1	106.8	---	---	-135.5
3 G	-116.1	-159.2	58.8	132.9	174.3	-84.4	-83.3
4 C	-83.2	54.5	168.5	132.5	-157.6	-73.2	-144.9
5 C	-61.0	167.2	42.9	112.0	-109.5	-67.2	-141.1
6 G	-90.5	68.9	178.9	86.9	-147.3	-80.8	-177.8
7 G	-68.8	-169.6	75.8	161.5	-139.1	-76.7	-88.7
8 C	-67.4	-176.0	38.6	87.3	173.1	-74.3	-150.0
9 G	-63.8	124.2	68.8	135.7	175.7	-86.8	-100.3
10 C	-69.3	-158.0	58.0	142.5	---	---	-110.7
11 G	-49.3	166.0	55.2	82.0	-165.8	-84.2	-154.2
12 C	-65.5	162.4	51.7	102.2	165.8	-79.7	-108.0
13 C	-82.4	-166.3	56.8	119.5	-152.7	-91.9	-112.9
14 G	-67.3	173.3	52.1	100.9	-172.7	-68.2	-156.6
15 G	-51.5	125.7	56.5	135.7	-152.1	-83.2	-137.1
16 C	-58.1	-174.3	38.7	145.0	-145.7	174.9	-95.6
17 G	-63.8	129.1	46.7	137.0	-171.3	-102.9	-94.5

S3 d. Table: Torsion angles parameters of the MD simulated structure at 300K and pH = 8 at 100.0 ns

Strand I

base	alpha	beta	gamma	delta	epsilon	zeta	chi
1 G	-92.4	62.9	170.1	145.0	-161.8	-111.8	-106.4
2 C	-56.6	154.2	63.3	105.9	-123.5	-79.3	-150.4
3 G	-107.3	76.1	149.9	155.3	174.5	-80.9	-139.0
4 G	-109.6	-119.3	48.6	152.7	-94.7	-72.8	-91.7
5 C	-77.9	-124.7	50.3	124.3	-178.7	-92.9	-136.3
6 C	-72.8	-158.7	48.4	77.4	-172.2	-60.7	-157.6
7 G	-69.2	-172.5	64.3	129.5	-159.7	-87.4	-112.8
8 C	-63.0	179.0	49.5	139.2	---	---	-89.5
9 G	-78.6	165.8	45.7	148.4	-177.9	-93.0	-79.0
10 C	-69.5	173.8	57.8	137.7	-97.0	174.2	-103.2
11 G	-70.6	130.0	41.3	124.5	168.3	-90.1	-127.4
12 G	-68.1	-143.8	39.6	158.5	-97.1	-95.8	-78.6
13 C	-64.3	-114.0	40.6	157.8	-134.3	-77.4	-147.2
14 C	-71.4	177.5	30.2	141.0	-129.7	-164.4	-97.7
15 G	-65.5	142.1	49.4	139.8	-157.2	-90.0	-134.6
16 C	-74.3	-165.7	28.9	143.9	---	---	-90.8

Strand II

base	alpha	beta	gamma	delta	epsilon	zeta	chi
1 C	-84.7	60.6	176.3	141.1	---	---	-157.8
2 G	-66.7	-158.0	31.0	160.4	-135.7	-88.9	-84.0
3 C	-83.6	166.1	45.7	102.5	-176.5	-85.9	-123.8
4 C	58.2	-79.5	-146.4	127.9	-157.9	-60.4	-146.4
5 G	-72.2	-174.2	51.6	97.3	-104.1	-160.6	-149.6
6 G	-65.2	162.1	52.6	130.5	-168.2	-86.9	-121.2
7 C	-60.5	138.6	64.5	102.0	-146.1	-83.3	-143.8
8 G	-69.6	176.9	79.9	145.6	-160.8	-131.7	-74.7
9 C	-81.7	-179.6	61.5	116.4	---	---	-109.9
10 G	-68.4	-153.7	44.5	156.7	-158.2	-90.9	-92.6
11 C	-57.9	145.8	61.1	87.8	-174.5	-78.2	-130.9
12 C	-50.6	170.2	56.7	143.4	-139.5	-117.9	-112.5
13 G	-57.0	122.3	55.1	86.9	-140.4	-77.9	-149.3
14 G	-64.2	173.5	39.6	133.6	-149.1	-168.0	-84.2
15 C	-66.6	143.7	70.5	142.5	-142.1	-92.7	-132.6
16 G	-76.3	81.5	-177.1	135.1	-162.1	-149.3	-108.2

S3 e. Table: Torsion angles parameters of the MD simulated structure at 300K and pH = 9 at 100.0 ns

Strand I

base	alpha	beta	gamma	delta	epsilon	zeta	chi
1 G	-78.0	152.9	52.9	121.1	-157.3	-79.1	-59.9
2 C	-79.0	47.5	-169.8	142.4	-169.3	-67.3	-131.6
3 G	-88.3	169.3	54.0	68.1	-156.3	-99.5	-147.6
4 G	-56.2	153.4	78.8	134.7	-70.8	-100.6	-113.4
5 C	-76.8	-126.0	60.3	149.1	-166.4	-82.9	-116.2
6 C	-70.2	175.7	50.5	116.0	-160.7	-101.8	-147.1
7 G	-153.1	-108.8	61.5	154.8	-124.1	176.6	-61.5
8 C	-74.2	127.3	61.9	143.2	---	---	-97.4
9 G	-55.5	114.7	55.6	156.2	-113.9	-147.1	-94.7
10 C	-71.2	141.3	51.6	141.8	-139.5	-152.2	-90.2
11 G	-62.2	130.4	66.4	92.4	-161.0	-83.3	-153.5
12 G	-73.9	-146.3	49.0	151.9	-61.5	-124.4	-91.4
13 C	-43.7	-121.6	49.8	144.5	-139.8	-84.8	-139.4
14 C	-77.3	170.0	42.8	139.5	-122.5	171.9	-79.4
15 G	-60.0	140.0	43.8	140.4	-158.2	-82.5	-140.0
16 C	-70.9	-178.3	42.6	131.1	---	---	-122.2

Strand II

base	alpha	beta	gamma	delta	epsilon	zeta	chi
1 C	-111.0	61.4	164.4	144.2	---	---	-136.7
2 G	-70.8	-150.0	53.9	152.2	-155.7	-64.7	-102.1
3 C	-106.9	69.5	156.3	97.2	-176.9	-79.3	-149.3
4 C	-54.6	172.9	51.5	142.3	-121.8	-66.1	-90.9
5 G	-86.3	-172.0	45.2	94.1	-161.8	-96.0	-138.9
6 G	-102.8	70.7	168.5	133.1	-144.9	-69.8	-162.4
7 C	-74.6	-174.1	44.7	142.1	-160.1	-64.2	-74.0
8 G	-61.1	140.9	47.7	151.3	-165.1	-94.7	-100.5
9 C	-42.9	160.7	48.8	136.8	---	---	-119.6
10 G	70.5	-130.9	-176.6	89.6	-134.8	-91.9	-153.1
11 C	-77.5	169.4	49.7	134.5	-91.6	163.9	-86.4
12 C	-114.9	75.3	171.4	131.9	-158.5	-60.8	-124.9
13 G	-63.3	-146.6	55.8	155.3	-145.4	-75.0	-88.6
14 G	-53.9	154.9	65.7	89.1	168.0	-78.9	-143.7
15 C	-53.3	-172.8	32.9	136.1	-164.9	-107.7	-108.6
16 G	-64.0	148.8	57.2	149.5	-173.4	-102.6	-119.5
