# Supplementary material for: Understanding the self-assembly dynamics of A/T absent ‘four-way DNA junctions with sticky ends’ at altered physiological conditions through molecular dynamics simulations
Source: PLoS One. 2023 Feb 8;18(2):e0278755. doi: 10.1371/journal.pone.0278755 (PMC9907842; doi:10.1371/journal.pone.0278755)
Supplement: S4 Table — (PDF) [file pone.0278755.s004.pdf]

Supporting information

Understanding the self-assembly dynamics of A/T absent 'four-way DNA junctions with sticky ends' at altered physiological conditions through molecular dynamics simulations

Akanksha Singh[1], Ramesh Kumar Yadav[2], Ali Shati[3], Nitin Kumar Kamboj[4], Hesham Hasssan[5,6], Shiv Bharadwaj[7*], Rashmi Rana[8*], Umesh Yadava[1*]

[1]Departmet of Physics, Deen Dayal Upadhyaya Gorakhpur University, Gorakhpur, 273009 India

[2]Department of Physics, B.R.D. Post Graduate College, Deoria, 274001 India

[3s]Department of Biology, Faculty of Science, King Khaild University, Abha, Saudi Arabia

[4]School of Physical Sciences, DIT University, Dehradun, UK, 248001, India

[5]Department of Pathology, College of Medicine, King Khaild University, Abha, Saudi Arabia

[6]Department of Pathology, Faculty of Medicine, Assiut University, Assiut, Egypt

[7]Department of Biotechnology, Institute of Biotechnology, College of Life and Applied Sciences, Yeungnam University, 280 Daehak-Ro, Gyeongsan, Gyeongbuk, 38541, Republic of Korea

[8]Department of Research, Sir Ganga Ram Hospital, New Delhi, India

*Corresponding authors

Email; SB: shiv@ynu.ac.kr

RR: Rashmi.rana@sgrh.com

UY: u_yadava@yahoo.com

S4 a. Table: Torsion angles parameters of the MD simulated structure at 310K and pH =5 at 100.0 ns

Strand I

base	alpha	beta	gamma	delta	epsilon	zeta	chi
1 G	-66.1	-176.0	52.4	144.7	-179.0	-84.0	-110.8
2 C	-68.5	166.7	69.0	95.6	-169.3	-83.3	-133.2
3 G	-74.0	-171.9	58.7	149.5	-95.5	174.3	-80.7
4 G	68.4	-106.8	-168.4	121.4	-90.1	-46.8	-132.1
5 C	-155.0	-112.8	78.6	73.3	86.4	73.2	-158.8
6 C	-159.4	-179.3	71.0	131.9	-147.7	-106.8	-108.2
7 G	-76.0	-178.8	47.1	156.6	-151.5	-167.1	-89.7
8 C	-40.6	130.0	46.5	136.0	---	---	-120.1
9 G	69.7	-93.6	-157.3	139.0	-153.9	-57.9	-152.3
10 C	-113.8	179.4	51.8	132.6	-143.1	-173.0	-77.4
11 G	-50.1	138.2	45.5	133.9	-168.3	-86.5	-105.7
12 G	-72.2	-177.4	64.7	145.0	-120.6	-66.8	-93.4
13 C	-62.5	-160.4	59.4	158.6	-122.7	-59.5	-166.6
14 C	-83.3	158.7	46.3	144.3	-142.6	-159.4	-52.4
15 G	-68.8	123.3	59.9	81.4	76.6	76.5	-148.8
16 C	-164.3	-169.9	73.7	127.8	---	---	-93.5

Strand II

base	alpha	beta	gamma	delta	epsilon	zeta	chi
1 C	69.5	-102.4	-144.7	112.0	---	---	-163.7
2 G	-94.2	158.7	65.7	138.6	-124.4	-169.9	-80.4
3 C	-125.2	74.6	162.6	164.9	-140.3	-98.9	-126.0
4 C	-64.1	162.5	56.9	138.8	-120.7	-63.6	-121.3
5 G	-67.3	169.8	63.5	108.7	-152.4	-135.4	-132.3
6 G	-54.6	134.6	43.2	139.8	-167.9	-81.5	-129.7
7 C	-64.5	-151.8	49.4	156.8	-130.3	-169.4	-77.2
8 G	-88.6	157.7	41.7	147.4	-179.0	-96.8	-114.1
9 C	175.0	162.5	172.8	133.0	---	---	-114.2
10 G	-73.0	-172.5	52.5	139.1	162.8	-82.9	-89.5
11 C	-57.8	142.7	53.5	143.1	-156.5	-122.1	-119.1
12 C	-58.5	178.7	46.9	143.6	-136.2	-165.9	-121.8
13 G	-67.3	159.6	38.8	146.2	-178.5	-96.7	-103.0
14 G	-53.4	137.5	42.3	157.0	-115.9	179.4	-102.7
15 C	-70.8	167.7	42.1	147.9	-121.6	178.7	-62.6
16 G	81.9	167.6	-174.0	127.3	-145.7	-86.3	-143.1

S4 b. Table: Torsion angles parameters of the MD simulated structure at 310K and pH =6 at 100.0 ns

Strand I

base	alpha	beta	gamma	delta	epsilon	zeta	chi
1 G	-66.1	148.1	50.8	158.8	-139.9	-108.6	-82.2
2 C	-71.8	156.1	52.3	130.5	-108.9	135.5	-96.4
3 G	-78.6	142.1	56.6	134.1	-162.2	-88.9	-140.9
4 G	-75.6	-148.4	42.0	152.2	-99.7	-89.9	-73.5
5 C	-71.1	-113.4	58.8	133.9	-119.3	-63.8	-179.5
6 C	-71.1	143.3	40.8	125.9	-149.0	-156.3	-82.3
7 G	-81.7	157.5	60.2	165.3	-153.6	-96.6	-121.5
8 C	-69.8	-172.0	27.7	142.5	---	---	-86.9
9 G	-90.3	58.0	172.3	103.2	-166.6	-91.0	-152.7
10 C	-71.4	-168.7	55.5	156.6	-134.6	-83.9	-86.7
11 G	-99.2	70.5	178.9	128.8	-106.5	-88.9	-169.6
12 G	-63.5	148.0	39.1	96.5	-130.6	-45.8	-118.8
13 C	-58.1	-120.9	62.5	160.5	-153.0	-66.8	-167.9
14 C	93.5	172.0	-167.4	140.8	-157.7	-89.5	-118.6
15 G	-74.6	166.9	57.1	90.2	-161.1	-74.5	-140.2
16 C	-65.3	168.1	53.8	115.9	---	---	-106.2

Strand II

base	alpha	beta	gamma	delta	epsilon	zeta	chi
1 C	-78.1	144.9	42.1	110.9	---	---	-63.5
2 G	-113.1	90.1	177.6	139.9	-104.1	-65.9	-177.6
3 C	-59.1	149.7	53.3	150.3	-162.1	-61.1	-101.3
4 C	-85.4	177.0	47.7	144.6	-145.0	-156.6	-88.6
5 G	-72.0	141.1	56.8	136.5	-154.4	-81.3	-129.2
6 G	-73.7	177.0	67.4	155.8	-118.9	-176.7	-98.6
7 C	-60.5	-178.5	39.0	106.5	-167.3	-93.2	-155.5
8 G	58.8	-140.3	-158.0	140.5	-178.4	-89.2	-100.2
9 C	-81.5	71.9	173.6	133.9	---	---	-152.1
10 G	-167.3	-128.4	76.4	106.0	-151.9	-83.4	-88.1
11 C	-65.4	172.0	73.0	125.7	-173.8	-89.1	-111.4
12 C	-69.9	-172.4	53.6	105.3	174.2	-87.7	-120.2
13 G	-70.8	159.8	67.6	90.9	-152.9	-70.7	-143.9
14 G	-48.0	148.9	44.5	141.4	-156.8	-90.8	-128.7
15 C	-39.8	109.0	39.0	130.4	-157.6	-152.4	-91.1
16 G	-64.0	166.7	63.3	136.3	-123.0	143.9	-61.2

S4 c. Table: Torsion angles parameters of the MD simulated structure at 310K and pH =7 at 100.0 ns

Strand I

base	alpha	beta	gamma	delta	epsilon	zeta	chi
1 G	-63.9	-163.3	66.9	139.3	-169.9	-101.8	-88.1
2 C	-45.0	148.1	48.4	94.2	-168.9	-98.1	-121.0
3 G	-68.7	175.5	69.5	147.6	171.0	-100.1	-88.7
4 G	-66.8	-143.5	39.2	165.3	-95.9	-83.5	-78.2
5 C	-91.8	-138.4	67.5	128.8	-143.2	-85.1	-160.5
6 C	-59.5	166.0	47.9	92.8	-160.9	-81.3	-152.9
7 G	-69.3	-175.3	62.8	163.7	-114.6	157.9	-86.1
8 C	-55.7	138.9	35.3	145.4	---	---	-109.3
9 G	-56.6	175.1	69.7	160.5	-172.8	-101.0	-75.2
10 C	-76.0	176.9	63.2	132.5	-128.8	-146.5	-106.2
11 G	-100.8	148.8	54.7	101.4	172.3	-95.4	-157.7
12 G	153.4	-145.0	-174.0	171.8	-83.2	-47.4	-143.2
13 C	-120.1	-104.3	58.7	128.1	-155.9	-82.4	-115.7
14 C	-73.4	176.5	44.3	116.2	177.7	-97.4	-151.0
15 G	-47.5	-161.1	50.1	140.8	-177.6	-91.6	-114.3
16 C	-78.7	-161.4	37.5	97.9	---	---	-113.0

Strand II

base	alpha	beta	gamma	delta	epsilon	zeta	chi
1 C	-68.0	174.7	59.0	119.1	---	---	-108.2
2 G	-64.1	168.0	56.2	115.8	-160.4	-96.5	-122.6
3 C	-71.5	176.5	45.7	84.5	-158.8	-93.9	-139.1
4 C	-56.2	-173.0	51.0	137.9	-169.4	-72.7	-107.3
5 G	-73.2	-152.8	46.4	137.7	179.2	-98.7	-120.7
6 G	-66.4	141.0	44.9	135.8	163.4	-83.2	-114.2
7 C	-72.9	172.7	42.0	140.0	-117.3	174.7	-98.6
8 G	-47.1	145.0	57.4	132.7	-159.1	-69.3	-113.2
9 C	-62.9	130.8	42.4	138.2	---	---	-117.2
10 G	-67.9	143.4	55.2	137.8	-112.4	175.8	-92.1
11 C	-54.3	169.5	64.1	135.6	-128.9	-100.0	-127.8
12 C	-80.4	-170.5	55.9	107.9	-179.2	-92.2	-138.0
13 G	-70.0	177.7	52.3	83.1	-167.8	-65.1	-160.2
14 G	-52.8	177.4	63.4	149.9	-153.5	-96.9	-125.4
15 C	-65.5	120.0	66.6	71.5	-147.8	-78.5	-153.9
16 G	-62.8	162.3	42.6	149.5	-142.7	-169.6	-60.2

S4 d. Table: Torsion angles parameters of the MD simulated structure at 310K and pH =8 at 100.0 ns

Strand I

base	alpha	beta	gamma	delta	epsilon	zeta	chi
1 G	-96.3	62.2	177.3	171.2	-163.4	-86.8	-66.7
2 C	-110.9	101.8	156.9	137.3	-95.1	-65.5	-146.0
3 G	-83.3	135.3	64.8	130.0	-132.1	165.0	-67.2
4 G	-61.8	120.7	66.5	137.3	-96.1	-67.0	-120.1
5 C	-93.0	-137.1	37.4	146.5	-142.1	-65.9	-165.2
6 C	-87.0	-176.3	42.6	138.5	-154.4	-67.5	-80.8
7 G	-90.0	46.0	-163.7	132.4	-112.1	-96.6	-158.3
8 C	-73.9	158.2	44.2	146.1	---	---	-62.8
9 G	-80.3	50.1	-175.4	135.3	-153.4	-74.0	-164.1
10 C	-71.6	169.4	58.6	126.6	-172.0	-112.2	-136.1
11 G	-66.1	-149.0	56.4	161.4	-169.0	-92.1	-75.2
12 G	-73.5	176.9	44.2	143.4	-90.9	-53.8	-79.9
13 C	-62.1	-142.8	51.0	151.3	-114.5	-79.7	-143.1
14 C	-59.1	140.3	48.1	129.1	-123.5	-172.8	-73.4
15 G	66.2	-111.8	177.2	95.7	-123.5	-79.5	-152.5
16 C	-69.4	169.6	56.7	158.1	---	---	-123.4

Strand II

base	alpha	beta	gamma	delta	epsilon	zeta	chi
1 C	-88.3	63.2	172.0	151.2	---	---	-149.5
2 G	-65.4	167.4	57.4	143.7	-159.3	-80.4	-68.3
3 C	-71.0	135.4	61.9	97.8	-169.2	-99.1	-97.4
4 C	-57.2	144.1	64.1	148.0	-91.3	-107.4	-116.8
5 G	-55.3	130.7	54.8	146.5	-149.3	-157.8	-115.6
6 G	-95.7	-173.4	50.2	140.3	-140.2	-172.5	-79.6
7 C	-86.8	63.7	169.8	120.8	-132.4	-81.1	-178.7
8 G	-57.3	125.5	51.9	138.9	-150.4	-78.2	-82.9
9 C	-70.6	-178.9	68.1	118.2	---	---	-136.3
10 G	93.5	-157.9	178.0	153.1	175.8	-98.0	-120.5
11 C	-82.0	149.8	53.2	129.9	-89.2	147.9	-59.7
12 C	-95.6	72.7	172.4	133.0	-106.3	-89.6	-157.2
13 G	-88.3	162.7	54.0	151.1	-141.0	-77.2	-75.8
14 G	-86.8	165.1	58.2	146.4	-110.2	179.6	-74.3
15 C	-100.5	67.7	166.6	138.2	-114.8	-64.5	-168.7
16 G	-60.3	124.4	60.5	136.5	-150.0	-90.6	-81.8

S4 e. Table: Torsion angles parameters of the MD simulated structure at 310K and pH =9 at 100.0 ns

Strand I

base	alpha	beta	gamma	delta	epsilon	zeta	chi
1 G	-54.5	131.0	51.6	142.5	-172.8	-100.5	-113.5
2 C	-68.6	-167.1	55.3	146.1	-136.0	-162.8	-95.6
3 G	-82.8	158.2	56.4	146.4	-175.8	-94.2	-107.7
4 G	-57.0	-161.8	34.7	134.3	-84.1	-70.6	-117.6
5 C	-89.6	-115.7	56.2	144.7	-152.3	-77.4	-141.8
6 C	-72.0	169.1	49.9	112.3	-177.6	-83.7	-120.0
7 G	-60.7	-163.0	39.5	144.9	-171.1	-85.5	-106.6
8 C	-62.1	-175.2	36.4	138.6	---	---	-86.8
9 G	-54.7	153.5	61.4	146.8	-94.0	166.0	-79.5
10 C	-77.4	124.7	59.2	122.3	-171.0	-107.1	-108.9
11 G	-57.3	171.5	55.0	86.6	-155.5	-83.1	-160.1
12 G	-78.4	-158.8	60.8	150.8	-81.4	-78.7	-85.3
13 C	-63.8	-134.9	50.9	148.6	-152.6	-86.5	-131.5
14 C	-68.3	161.1	60.4	94.6	-167.7	-89.3	-151.2
15 G	-71.3	-148.4	46.3	149.5	178.5	-105.7	-98.1
16 C	-62.5	-173.1	49.6	157.0	---	---	-95.2

Strand II

base	alpha	beta	gamma	delta	epsilon	zeta	chi
1 C	-69.6	127.7	61.8	122.1	---	---	-142.4
2 G	-64.0	167.8	43.6	151.0	-116.7	169.7	-87.3
3 C	-47.2	173.7	61.6	113.4	-159.7	-73.1	-117.1
4 C	-60.0	172.1	51.6	118.7	172.1	-88.9	-120.6
5 G	-65.3	-165.6	51.7	113.0	-149.3	-87.9	-141.1
6 G	-103.6	127.9	59.9	78.0	-169.3	-59.2	-163.7
7 C	-76.4	142.8	52.5	138.3	-113.3	-156.6	-90.9
8 G	-60.8	125.0	45.4	155.8	-114.9	-151.8	-88.3
9 C	-76.0	164.0	46.3	132.9	---	---	-72.7
10 G	-65.2	-176.7	49.4	111.5	-143.5	-68.3	-146.7
11 C	-62.6	119.1	56.2	123.9	-170.3	-90.6	-122.5
12 C	-69.6	167.6	33.4	139.7	-108.8	170.8	-104.2
13 G	-88.0	-123.8	44.6	156.2	-153.4	-94.4	-92.6
14 G	-108.1	66.0	178.2	145.3	174.3	-92.9	-144.9
15 C	-58.4	169.2	46.9	133.8	-154.2	-57.0	-105.0
16 G	-62.2	141.6	70.4	154.9	-167.2	-96.4	-108.3
