# Supplementary material for: Understanding the self-assembly dynamics of A/T absent ‘four-way DNA junctions with sticky ends’ at altered physiological conditions through molecular dynamics simulations
Source: PLoS One. 2023 Feb 8;18(2):e0278755. doi: 10.1371/journal.pone.0278755 (PMC9907842; doi:10.1371/journal.pone.0278755)
Supplement: S5 Table — (PDF) [file pone.0278755.s005.pdf]

Supporting information

Understanding the self-assembly dynamics of A/T absent 'four-way DNA junctions with sticky ends' at altered physiological conditions through molecular dynamics simulations

Akanksha Singh[1], Ramesh Kumar Yadav[2], Ali Shati[3], Nitin Kumar Kamboj[4], Hesham Hasssan[5,6], Shiv Bharadwaj[7*], Rashmi Rana[8*], Umesh Yadava[1*]

[1]Departmet of Physics, Deen Dayal Upadhyaya Gorakhpur University, Gorakhpur, 273009 India

[2]Department of Physics, B.R.D. Post Graduate College, Deoria, 274001 India

[3s]Department of Biology, Faculty of Science, King Khaild University, Abha, Saudi Arabia

[4]School of Physical Sciences, DIT University, Dehradun, UK, 248001, India

[5]Department of Pathology, College of Medicine, King Khaild University, Abha, Saudi Arabia

[6]Department of Pathology, Faculty of Medicine, Assiut University, Assiut, Egypt

[7]Department of Biotechnology, Institute of Biotechnology, College of Life and Applied Sciences, Yeungnam University, 280 Daehak-Ro, Gyeongsan, Gyeongbuk, 38541, Republic of Korea

[8]Department of Research, Sir Ganga Ram Hospital, New Delhi, India

*Corresponding authors

Email; SB: shiv@ynu.ac.kr

RR: Rashmi.rana@sgrh.com

UY: u_yadava@yahoo.com

S5 a. Table: Local base-pair, local base-pair step and local base-pair helical parameters of the MD simulated structure at 300K and pH =5

bp	Shear	Stretch	Stagger	Buckle	Propeller	Opening
1 G-C	-0.47	-0.27	-0.59	-3.51	0.46	1.39
2 C-G	0.27	-0.04	-0.15	5.20	-19.25	5.11
3 G-C	-0.34	-0.13	0.02	-0.07	-13.56	-6.88
4 G-C	0.05	-0.14	-0.23	-1.06	-2.28	2.49
5 C-G	-0.34	-0.15	-0.55	10.07	2.56	-1.93
6 C-G	0.40	-0.24	0.81	-22.01	-20.35	-3.02
7 G-C	0.01	-0.03	0.34	-13.06	-7.56	1.60
8 C-G	-0.02	-0.13	-0.19	6.84	6.08	0.50
9 G-C	-0.51	-0.25	0.12	2.86	-5.67	-5.60
10 C-G	-0.11	-0.11	0.06	15.48	-5.23	-2.85
11 G-C	0.04	-0.07	0.02	-3.69	-17.62	-0.28
12 G-C	0.09	-0.15	0.33	18.02	-21.43	-1.54
13 C-G	-0.32	-0.19	-0.52	7.90	-17.83	-6.41
14 C-G	0.18	-0.20	-0.08	11.98	-8.44	-0.76
15 G-C	-0.24	-0.07	-0.04	-6.25	-15.50	-6.44
16 C-G	0.69	-0.01	0.49	0.77	8.64	9.41

step	Shift	Slide	Rise	Tilt	Roll	Twist
1 GC/GC	-0.52	-0.03	3.00	-4.99	10.25	33.84
2 CG/CG	-1.06	-1.08	3.33	-7.56	17.69	28.91
3 GG/CC	0.24	0.13	3.61	7.28	-4.76	43.11
4 GC/GC	-0.47	-0.14	3.16	-0.79	0.97	25.22
5 CC/GG	0.89	-2.04	4.35	-1.82	0.48	35.16
6 CG/CG	-0.25	-0.84	3.00	2.60	11.02	31.36
7 GC/GC	0.17	0.22	2.89	4.11	3.54	34.65
8 CG/CG	----	----	----	----	----	----
9 GC/GC	0.03	-0.03	3.13	0.51	7.29	32.97
10 CG/CG	0.92	-0.59	3.87	3.56	-1.75	32.61
11 GG/CC	-0.33	0.92	2.81	-2.45	2.79	40.27
12 GC/GC	-0.75	-0.52	3.55	5.71	2.76	24.80
13 CC/GG	1.10	1.41	3.29	3.57	10.04	40.38
14 CG/CG	-1.04	1.91	3.86	-4.60	1.42	36.85
15 GC/GC	1.57	-0.30	3.18	-5.43	3.13	36.00

step	X-disp	Y-disp	h-Rise	Incl.	Tip	h-Twist
1 GC/GC	-1.43	0.18	2.91	17.02	8.29	35.66
2 CG/CG	-4.46	0.64	2.48	31.43	13.43	34.62
3 GG/CC	0.68	0.44	3.57	-6.40	-9.78	43.94
4 GC/GC	-0.60	0.84	3.17	2.21	1.81	25.25
5 CC/GG	-3.47	-1.83	4.28	0.79	3.02	35.21
6 CG/CG	-3.08	0.82	2.54	19.61	-4.62	33.29
7 GC/GC	-0.11	0.27	2.90	5.89	-6.85	35.06
8 CG/CG	----	----	----	----	----	----
9 GC/GC	-1.19	0.03	3.05	12.65	-0.89	33.75
10 CG/CG	-0.69	-0.89	3.97	-3.11	-6.31	32.84
11 GG/CC	1.06	0.23	2.88	4.05	3.55	40.44
12 GC/GC	-2.00	3.40	3.22	6.30	-13.03	25.58
13 CC/GG	0.85	-1.14	3.61	14.25	-5.07	41.71
14 CG/CG	2.77	0.89	4.03	2.23	7.23	37.15
15 GC/GC	-0.88	-3.21	2.89	5.02	8.71	36.52

S5 b. Table: Local base-pair, local base-pair step and local base-pair helical parameters of the MD simulated structure at 300K and pH = 6

bp	Shear	Stretch	Stagger	Buckle	Propeller	Opening
1 G-C	-0.10	-0.14	0.20	3.68	-0.47	2.49
2 C-G	0.27	-0.09	0.80	-24.64	-2.06	2.21
3 G-C	-0.41	-0.30	0.21	0.59	5.11	-3.37
4 G-C	0.25	-0.21	0.03	2.41	-17.42	0.97
5 C-G	0.20	-0.22	-0.34	3.48	-26.16	4.24
6 C-G	0.08	-0.31	-0.07	-1.50	-18.48	-1.72
7 G-C	0.66	0.15	0.51	-14.52	-27.71	-12.35
8 C-G	0.49	-0.14	-0.35	1.74	15.48	-1.22
9 G-C	-0.51	-0.38	0.40	8.25	-16.65	-3.08
10 C-G	0.23	-0.38	0.03	-2.20	-23.90	0.35
11 G-C	-0.53	-0.04	-0.69	-28.93	-11.59	4.40
12 G-C	0.02	-0.25	-0.11	-12.64	-11.27	-2.83
13 C-G	0.07	-0.22	-0.11	9.02	-14.96	-3.16
14 C-G	0.50	-0.21	-0.84	20.44	1.73	2.03
15 G-C	-0.58	-0.10	0.29	17.22	9.78	3.03
16 C-G	0.15	-0.01	-0.58	8.08	-7.63	5.04

step	Shift	Slide	Rise	Tilt	Roll	Twist
1 GC/GC	-1.13	-1.86	4.26	-5.40	0.88	38.29
2 CG/CG	-0.94	1.31	2.92	3.84	-4.34	41.13
3 GG/CC	-0.98	1.01	3.49	-4.86	12.04	32.25
4 GC/GC	0.60	-0.05	3.27	7.36	-1.81	30.84
5 CC/GG	-0.09	1.64	3.65	2.68	2.51	38.46
6 CG/CG	0.48	-0.36	3.63	-3.78	7.29	40.40
7 GC/GC	1.60	0.18	3.10	9.43	1.90	30.99
8 CG/CG	----	----	----	----	----	----
9 GC/GC	0.84	-0.79	3.63	5.90	-0.82	43.90
10 CG/CG	1.12	0.55	4.06	6.54	11.91	31.52
11 GG/CC	-1.29	0.79	3.04	-7.34	-0.78	37.78
12 GC/GC	2.04	-0.48	2.99	4.13	2.45	27.13
13 CC/GG	1.39	1.72	3.37	12.96	-0.77	37.19
14 CG/CG	0.00	0.91	3.68	-9.61	18.57	33.91
15 GC/GC	-0.83	-0.73	3.70	5.59	3.83	34.54

step	X-disp	Y-disp	h-Rise	Incl.	Tip	h-Twist
1 GC/GC	-2.95	0.83	4.34	1.33	8.19	38.66
2 CG/CG	2.25	1.68	2.67	-6.15	-5.43	41.52
3 GG/CC	-0.43	0.79	3.73	20.66	8.35	34.70
4 GC/GC	0.25	0.29	3.32	-3.34	-13.59	31.73
5 CC/GG	2.13	0.50	3.73	3.80	-4.06	38.63
6 CG/CG	-1.38	-1.14	3.46	10.42	5.40	41.20
7 GC/GC	-0.02	-1.17	3.43	3.44	-17.13	32.42
8 CG/CG	----	----	----	----	----	----
9 GC/GC	-0.97	-0.50	3.72	-1.09	-7.85	44.28
10 CG/CG	-1.51	-0.55	4.14	20.76	-11.39	34.26
11 GG/CC	1.30	1.07	3.21	-1.19	11.21	38.47
12 GC/GC	-1.58	-3.32	3.20	5.18	-8.71	27.55
13 CC/GG	2.66	-0.37	3.60	-1.17	-19.60	39.32
14 CG/CG	-1.27	-1.35	3.57	28.70	14.85	39.67
15 GC/GC	-1.83	2.30	3.43	6.37	-9.31	35.17

S5 c. Table: Local base-pair, local base-pair step and local base-pair helical parameters of the MD simulated structure at 300K and pH = 7

bp	Shear	Stretch	Stagger	Buckle	Propeller	Opening
1 G-C	-1.82	6.80	0.85	52.37	21.29	163.48
2 G-C	-0.44	-0.21	0.39	6.77	-1.43	-0.86
3 C-G	-0.06	0.01	0.56	-7.74	-14.74	-1.97
4 G-C	-0.14	-0.20	-0.52	-14.13	-32.17	-3.02
5 G-C	-0.88	-0.42	-0.19	-8.57	-32.92	1.38
6 C-G	-0.02	-0.31	0.28	-19.08	-20.79	-8.89
7 C-G	-0.13	0.05	-0.31	-11.57	-0.49	2.50
8 G-C	-0.55	0.19	0.13	10.32	-6.73	10.16
9 C-G	-0.22	-0.06	-0.84	25.84	7.37	0.96
10 G-C	-0.15	-0.16	0.07	-4.38	12.43	-2.82
11 C-G	0.09	-0.36	0.63	1.53	-13.34	-6.21
12 G-C	-0.12	0.04	-0.31	-2.01	0.65	5.27
13 G-C	0.41	-0.17	-0.07	13.84	-9.80	0.26
14 C-G	0.94	-0.25	0.33	5.61	-21.98	5.78
15 C-G	0.11	-0.10	-0.11	-11.50	-12.49	-2.15
16 G-C	0.02	-0.22	-0.25	-7.31	-1.22	-1.78
17 C-G	-0.21	-0.21	-0.42	11.64	23.10	-3.29

step	Shift	Slide	Rise	Tilt	Roll	Twist
1 GG/CC	-0.03	3.54	2.60	24.51	-19.57	113.89
2 GC/GC	-0.38	-1.00	3.89	-0.04	-2.12	23.71
3 CG/CG	0.30	0.16	3.57	6.58	11.32	38.38
4 GG/CC	0.23	0.61	3.37	-4.44	2.26	25.35
5 GC/GC	0.50	-1.36	3.67	0.16	-0.67	40.62
6 CC/GG	-0.83	-0.57	3.31	2.15	1.01	27.21
7 CG/CG	0.52	0.17	3.01	-9.86	6.29	22.61
8 GC/GC	-0.70	0.17	3.32	9.15	-1.04	33.05
9 CG/CG	----	----	----	----	----	----
10 GC/GC	0.97	-0.89	3.57	-4.84	-1.53	27.76
11 CG/CG	-0.05	-1.07	3.47	7.97	3.52	28.38
12 GG/CC	-1.10	1.22	3.11	-1.39	3.64	38.30
13 GC/GC	0.06	-0.67	3.49	-3.21	8.99	43.13
14 CC/GG	-0.79	-2.07	3.80	10.05	7.04	29.90
15 CG/CG	1.76	-0.13	3.44	5.28	-4.46	24.09
16 GC/GC	-0.30	-0.49	3.43	0.57	11.98	24.21

step	X-disp	Y-disp	h-Rise	Incl.	Tip	h-Twist
1 GG/CC	2.23	0.21	2.18	-11.55	-14.47	116.65
2 GC/GC	-1.59	0.90	3.96	-5.15	0.11	23.81
3 CG/CG	-1.18	0.39	3.48	16.64	-9.67	40.48
4 GG/CC	0.71	-1.79	3.32	5.08	10.00	25.83
5 GC/GC	-1.88	-0.70	3.69	-0.97	-0.24	40.63
6 CC/GG	-1.45	2.29	3.21	2.14	-4.56	27.31
7 CG/CG	-1.23	-3.74	2.53	14.84	23.25	25.42
8 GC/GC	0.45	2.60	3.02	-1.78	-15.71	34.27
9 CG/CG	----	----	----	----	----	----
10 GC/GC	-1.45	-3.20	3.40	-3.15	9.99	28.21
11 CG/CG	-2.86	1.85	3.19	6.97	-15.79	29.66
12 GG/CC	1.41	1.50	3.24	5.53	2.12	38.49
13 GC/GC	-1.77	-0.39	3.28	12.05	4.30	44.13
14 CC/GG	-5.05	3.31	2.84	12.98	-18.52	32.26
15 CG/CG	1.17	-2.32	3.70	-10.40	-12.33	25.05
16 GC/GC	-4.14	0.80	2.86	26.58	-1.26	26.98

S5 d. Table: Local base-pair, local base-pair step and local base-pair helical parameters of the MD simulated structure at 300K and pH = 8

bp	Shear	Stretch	Stagger	Buckle	Propeller	Opening
1 G-C	-0.22	-0.16	-0.24	-11.81	-10.40	-0.62
2 C-G	0.28	-0.25	0.70	-10.08	-1.47	0.15
3 G-C	-0.06	-0.24	-0.21	2.01	-7.57	0.59
4 G-C	-0.48	-0.23	-0.53	-21.83	-13.63	2.92
5 C-G	0.10	-0.06	-0.34	6.94	2.23	1.42
6 C-G	0.44	-0.29	0.12	-15.37	-19.88	-2.21
7 G-C	-0.05	-0.23	0.07	-12.22	-19.69	-0.32
8 C-G	0.50	-0.20	-0.81	8.39	0.41	0.17
9 G-C	-0.09	-0.38	-0.70	-11.85	7.98	-1.56
10 C-G	0.02	0.01	-0.39	-6.03	1.93	-1.93
11 G-C	-0.14	0.03	-0.20	-14.41	-10.95	3.48
12 G-C	0.35	-0.21	-0.53	-6.58	-6.87	-1.08
13 C-G	-0.22	-0.13	0.36	-3.19	-37.38	-0.84
14 C-G	0.25	-0.17	-0.46	-12.70	-5.46	2.01
15 G-C	-0.03	-0.34	0.40	-6.24	-6.94	-3.23
16 C-G	-0.06	-0.09	0.00	1.20	19.17	4.30

step	Shift	Slide	Rise	Tilt	Roll	Twist
1 GC/GC	-0.18	-0.59	3.32	-8.44	0.59	29.31
2 CG/CG	0.22	0.09	3.19	8.74	-3.08	30.39
3 GG/CC	-0.66	-0.86	4.00	1.60	6.76	42.39
4 GC/GC	0.71	0.38	2.83	-7.06	3.37	22.34
5 CC/GG	-0.96	-1.99	4.14	1.89	-8.31	35.91
6 CG/CG	0.12	-0.28	3.22	2.05	16.61	28.44
7 GC/GC	0.38	0.89	2.78	7.81	1.25	35.55
8 CG/CG	----	----	----	----	----	----
9 GC/GC	-0.45	-0.15	3.41	-2.30	1.07	19.69
10 CG/CG	-0.00	0.80	3.76	-1.47	0.36	39.16
11 GG/CC	-0.67	0.06	3.29	0.92	8.55	39.10
12 GC/GC	0.24	-0.14	3.41	-3.64	-2.16	28.59
13 CC/GG	0.77	0.28	3.57	9.67	6.12	40.76
14 CG/CG	-1.40	0.45	3.62	-16.73	0.80	31.49
15 GC/GC	1.35	-0.21	3.39	4.14	2.37	32.85

step	X-disp	Y-disp	h-Rise	Incl.	Tip	h-Twist
1 GC/GC	-1.24	-1.38	3.24	1.13	16.26	30.48
2 CG/CG	0.72	1.18	3.11	-5.71	-16.21	31.74
3 GG/CC	-1.97	1.10	3.80	9.27	-2.19	42.93
4 GC/GC	-0.02	-3.71	2.52	8.38	17.54	23.66
5 CC/GG	-1.65	1.85	4.42	-13.26	-3.01	36.87
6 CG/CG	-3.24	0.13	2.66	30.68	-3.79	32.91
7 GC/GC	1.27	0.33	2.83	2.01	-12.60	36.39
8 CG/CG	----	----	----	----	----	----
9 GC/GC	-0.96	0.18	3.42	3.10	6.70	19.85
10 CG/CG	1.14	-0.20	3.76	0.54	2.20	39.18
11 GG/CC	-0.90	1.09	3.21	12.59	-1.35	40.00
12 GC/GC	0.23	-1.32	3.36	-4.34	7.31	28.89
13 CC/GG	-0.32	0.05	3.65	8.58	-13.57	42.27
14 CG/CG	0.60	-0.68	3.87	1.37	28.45	35.57
15 GC/GC	-0.79	-1.63	3.51	4.16	-7.27	33.19

S5 e. Table: Local base-pair, local base-pair step and local base-pair helical parameters of the MD simulated structure at 300K and pH = 9

bp	Shear	Stretch	Stagger	Buckle	Propeller	Opening
1 G-C	-0.24	-0.17	-0.67	-9.06	-17.52	1.84
2 C-G	-0.06	-0.13	-0.17	1.87	-23.18	-8.09
3 G-C	0.14	-0.20	-0.17	-17.65	-22.43	3.43
4 G-C	-0.68	-0.21	-0.07	2.23	-14.35	0.97
5 C-G	0.61	-0.33	-0.67	26.82	-17.26	-4.25
6 C-G	0.22	-0.35	-0.32	8.93	-17.53	-1.31
7 G-C	0.02	-0.13	-0.90	-5.69	4.62	0.31
8 C-G	0.18	-0.35	-0.01	16.36	-6.67	-8.16
9 G-C	-0.46	-0.36	-0.30	1.10	9.25	1.09
10 C-G	-0.05	0.05	-0.10	23.11	-20.12	4.85
11 G-C	-0.56	-0.10	0.53	-5.81	-17.69	5.19
12 G-C	-0.12	-0.03	0.10	-7.44	-16.28	-2.25
13 C-G	-0.11	-0.08	0.36	10.60	-16.35	-1.15
14 C-G	0.03	-0.04	-0.62	40.54	-17.37	0.21
15 G-C	-0.42	-0.35	0.47	6.19	-17.54	0.68
16 C-G	0.26	-0.28	0.24	-5.86	-0.10	-4.48
4 GC/GC	0.31	0.83	2.83	9.03	4.73	39.29
5 CC/GG	0.48	-2.56	3.79	-6.51	11.58	28.66
6 CG/CG	-1.41	-0.41	3.88	5.83	15.36	22.99
7 GC/GC	-1.27	0.64	2.78	-10.90	6.71	29.59
8 CG/CG	----	----	----	----	----	----
9 GC/GC	-0.14	0.51	2.93	3.91	2.89	34.67
10 CG/CG	0.14	-0.35	4.04	-4.05	1.90	39.74
11 GG/CC	-1.93	0.29	3.72	0.48	14.03	36.47
12 GC/GC	-0.03	0.36	3.04	-4.64	-5.54	26.73
13 CC/GG	0.80	0.37	2.98	4.62	1.93	32.93
14 CG/CG	-0.90	-0.20	4.60	-13.51	-2.76	36.04
15 GC/GC	-0.29	-0.89	3.72	-0.85	1.26	41.24

step	X-disp	Y-disp	h-Rise	Incl.	Tip	h-Twist
1 GC/GC	2.09	-0.24	2.87	-8.02	2.80	24.86
2 CG/CG	-1.42	-2.20	3.79	12.65	-4.33	42.27
3 GG/CC	2.77	1.71	2.92	-5.07	-5.52	27.26
4 GC/GC	0.73	0.45	2.91	6.89	-13.17	40.54
5 CC/GG	-6.86	-2.09	2.44	21.97	12.36	31.53
6 CG/CG	-4.81	4.40	2.67	33.59	-12.74	28.19
7 GC/GC	0.03	0.47	3.11	12.43	20.19	32.19
8 CG/CG	----	----	----	----	----	----
9 GC/GC	0.45	0.76	2.93	4.81	-6.53	35.00
10 CG/CG	-0.78	-0.78	3.99	2.78	5.93	39.98
11 GG/CC	-1.53	2.96	3.56	21.46	-0.73	39.00
12 GC/GC	1.98	-0.97	2.87	-11.71	9.81	27.67
13 CC/GG	0.35	-0.68	3.08	3.37	-8.09	33.30
14 CG/CG	0.19	-1.08	4.63	-4.27	20.91	38.51
15 GC/GC	-1.42	0.30	3.69	1.79	1.21	41.27
