# Supplementary material for: Understanding the self-assembly dynamics of A/T absent ‘four-way DNA junctions with sticky ends’ at altered physiological conditions through molecular dynamics simulations
Source: PLoS One. 2023 Feb 8;18(2):e0278755. doi: 10.1371/journal.pone.0278755 (PMC9907842; doi:10.1371/journal.pone.0278755)
Supplement: S6 Table — (PDF) [file pone.0278755.s006.pdf]

Supporting information

Understanding the self-assembly dynamics of A/T absent 'four-way DNA junctions with sticky ends' at altered physiological conditions through molecular dynamics simulations

Akanksha Singh[1], Ramesh Kumar Yadav[2], Ali Shati[3], Nitin Kumar Kamboj[4], Hesham Hasssan[5,6], Shiv Bharadwaj[7*], Rashmi Rana[8*], Umesh Yadava[1*]

[1]Departmet of Physics, Deen Dayal Upadhyaya Gorakhpur University, Gorakhpur, 273009 India

[2]Department of Physics, B.R.D. Post Graduate College, Deoria, 274001 India

[3s]Department of Biology, Faculty of Science, King Khaild University, Abha, Saudi Arabia

[4]School of Physical Sciences, DIT University, Dehradun, UK, 248001, India

[5]Department of Pathology, College of Medicine, King Khaild University, Abha, Saudi Arabia

[6]Department of Pathology, Faculty of Medicine, Assiut University, Assiut, Egypt

[7]Department of Biotechnology, Institute of Biotechnology, College of Life and Applied Sciences, Yeungnam University, 280 Daehak-Ro, Gyeongsan, Gyeongbuk, 38541, Republic of Korea

[8]Department of Research, Sir Ganga Ram Hospital, New Delhi, India

*Corresponding authors

Email; SB: shiv@ynu.ac.kr

RR: Rashmi.rana@sgrh.com

UY: u_yadava@yahoo.com

S6 a. Table: Local base-pair, local base-pair step and local base-pair helical parameters of the MD simulated structure at 310K and pH =5

bp	Shear	Stretch	Stagger	Buckle	Propeller	Opening
1 G-C	0.05	-0.28	-0.16	-16.30	-26.50	-4.03
2 C-G	0.07	0.32	0.13	-6.70	-14.48	9.11
3 G-C	-0.04	-0.20	0.26	-0.28	-22.67	-4.84
4 G-C	-0.09	-0.05	-0.09	-6.41	-26.97	0.56
5 C-G	-0.19	0.00	0.83	-0.55	-30.58	3.10
6 C-G	-0.37	-0.14	0.05	4.00	-12.87	-2.53
7 G-C	0.08	0.02	-0.08	-2.78	-4.61	7.50
8 C-G	-0.23	-0.02	-0.21	10.49	-16.00	-0.29
9 G-C	-0.53	-0.24	-0.38	-3.71	-20.01	2.19
10 C-G	0.76	-0.26	-0.20	1.67	-14.57	3.23
11 G-C	0.08	-0.17	0.14	-4.97	-17.39	-7.32
12 G-C	0.04	-0.04	0.58	15.80	-4.52	-7.64
13 C-G	0.11	-0.14	-0.16	16.75	-11.46	-4.20
14 C-G	0.02	-0.12	-0.79	31.74	-22.95	-1.04
15 G-C	0.04	-0.12	-0.80	-27.28	-31.01	0.77
16 C-G	0.17	-0.33	0.02	-1.43	-1.10	-9.10

step	Shift	Slide	Rise	Tilt	Roll	Twist
1 GC/GC	0.65	-0.26	2.95	0.26	-4.22	30.08
2 CG/CG	-0.34	0.89	3.60	4.21	-0.64	36.07
3 GG/CC	0.29	1.64	3.79	2.06	5.91	36.20
4 GC/GC	1.94	-0.40	3.05	4.06	-3.81	35.49
5 CC/GG	-1.03	-1.28	3.15	4.81	15.08	30.51
6 CG/CG	1.08	1.27	3.62	5.29	-4.40	39.54
7 GC/GC	-0.09	0.33	3.18	0.74	-2.58	37.27
8 CG/CG	----	----	----	----	----	----
9 GC/GC	0.14	0.07	3.12	-2.89	8.66	36.69
10 CG/CG	-0.27	1.46	3.77	-2.51	5.67	43.80
11 GG/CC	0.77	-0.04	2.82	0.59	4.08	31.63
12 GC/GC	0.59	-0.94	3.52	3.79	-1.30	24.83
13 CC/GG	0.46	1.77	2.76	13.66	-2.68	46.40
14 CG/CG	-0.11	2.19	4.98	-4.28	11.71	42.12
15 GC/GC	-0.59	1.10	2.68	-9.93	11.52	35.28

step	X-disp	Y-disp	h-Rise	Incl.	Tip	h-Twist
1 GC/GC	0.27	-1.19	2.96	-8.08	-0.50	30.37
2 CG/CG	1.52	1.18	3.53	-1.03	-6.76	36.32
3 GG/CC	1.63	-0.13	4.01	9.43	-3.28	36.72
4 GC/GC	-0.12	-2.58	3.27	-6.21	-6.61	35.91
5 CC/GG	-4.20	2.40	2.13	26.53	-8.46	34.28
6 CG/CG	2.40	-0.91	3.57	-6.45	-7.75	40.11
7 GC/GC	0.85	0.24	3.15	-4.04	-1.16	37.36
8 CG/CG	----	----	----	----	----	----
9 GC/GC	-0.96	-0.58	3.04	13.51	4.50	37.77
10 CG/CG	1.32	0.08	3.93	7.56	3.35	44.22
11 GG/CC	-0.71	-1.30	2.81	7.44	-1.08	31.89
12 GC/GC	-1.74	-0.13	3.62	-3.00	-8.75	25.15
13 CC/GG	2.34	0.34	2.68	-3.31	-16.89	48.34
14 CG/CG	1.13	-0.51	5.36	15.88	5.80	43.85
15 GC/GC	0.37	-0.22	2.94	18.01	15.52	38.32

S6 b. Table: Local base-pair, local base-pair step and local base-pair helical parameters of the MD simulated structure at 310K and pH =6

bp	Shear	Stretch	Stagger	Buckle	Propeller	Opening
1 G-C	-0.73	-0.18	-0.62	-20.56	-2.63	-0.94
2 C-G	0.20	-0.18	-0.04	4.96	0.87	-3.07
3 G-C	-0.10	-0.04	0.44	1.75	-12.04	-7.41
4 G-C	-0.68	-0.24	-0.17	2.33	-4.32	-0.47
5 C-G	0.34	-0.27	0.40	2.16	-19.67	-5.47
6 C-G	-0.18	-0.11	-0.61	13.57	-2.01	-0.12
7 G-C	-0.35	-0.12	0.42	17.56	7.18	-2.17
8 C-G	0.24	-0.25	-0.37	13.84	-4.66	-1.05
9 G-C	-0.26	-0.30	-0.24	-14.14	-4.79	-2.47
10 C-G	-0.40	-0.13	-0.06	9.20	-10.24	0.04
11 G-C	-0.19	-0.29	-0.34	-10.18	-29.17	6.04
12 G-C	-0.32	-0.18	-0.22	-8.59	-10.01	2.50
13 C-G	0.14	0.12	0.20	-9.36	-27.39	7.78
14 C-G	-0.21	-0.19	-0.15	-8.44	2.61	-1.75
15 G-C	0.12	-0.01	-0.09	-11.97	3.85	2.42
16 C-G	0.59	-0.26	-0.43	6.34	26.62	6.58

step	Shift	Slide	Rise	Tilt	Roll	Twist
1 GC/GC	0.65	0.46	2.78	-6.02	6.69	24.75
2 CG/CG	-1.97	-0.94	3.56	-6.44	-5.08	27.57
3 GG/CC	-0.03	0.79	3.60	3.41	0.52	39.49
4 GC/GC	1.06	-0.09	3.37	-0.47	-0.83	29.88
5 CC/GG	1.19	1.02	3.17	12.99	-2.15	37.62
6 CG/CG	-0.66	0.50	3.36	-12.91	9.14	37.36
7 GC/GC	-0.30	-1.08	3.72	6.45	11.50	30.32
8 CG/CG	----	----	----	----	----	----
9 GC/GC	-1.04	-0.15	2.90	-2.17	3.21	22.05
10 CG/CG	0.57	-0.67	4.14	3.49	5.08	19.71
11 GG/CC	-0.48	-0.50	3.28	-0.19	4.55	39.08
12 GC/GC	1.42	-1.48	3.33	-0.11	14.70	22.59
13 CC/GG	-1.24	-1.12	3.22	-1.42	10.08	32.51
14 CG/CG	0.86	-0.49	3.63	-1.47	4.04	32.45
15 GC/GC	0.78	0.38	3.22	0.84	6.44	26.44

step	X-disp	Y-disp	h-Rise	Incl.	Tip	h-Twist
1 GC/GC	-0.51	-2.79	2.59	15.00	13.48	26.31
2 CG/CG	-0.55	2.26	4.01	-10.36	13.15	28.74
3 GG/CC	1.09	0.48	3.60	0.76	-5.04	39.64
4 GC/GC	-0.01	-2.15	3.36	-1.60	0.91	29.89
5 CC/GG	1.76	-0.20	3.32	-3.21	-19.43	39.78
6 CG/CG	-0.38	-0.62	3.43	13.55	19.14	40.46
7 GC/GC	-4.00	1.71	3.00	20.80	-11.66	33.00
8 CG/CG	----	----	----	----	----	----
9 GC/GC	-1.46	1.95	2.93	8.31	5.62	22.39
10 CG/CG	-4.66	0.33	3.88	14.39	-9.90	20.64
11 GG/CC	-1.29	0.70	3.21	6.78	0.28	39.33
12 GC/GC	-6.53	-3.08	2.00	33.37	0.24	26.90
13 CC/GG	-3.44	1.91	2.81	17.48	2.46	34.03
14 CG/CG	-1.64	-1.80	3.50	7.20	2.62	32.73
15 GC/GC	-0.81	-1.44	3.24	13.82	-1.81	27.21

S6 c. Table: Local base-pair, local base-pair step and local base-pair helical parameters of the MD simulated structure at 310K and pH =7

bp	Shear	Stretch	Stagger	Buckle	Propeller	Opening
1 G-C	-0.50	-0.24	-0.20	-12.11	1.90	-0.70
2 C-G	0.12	-0.03	0.09	9.68	-1.90	3.06
3 G-C	-0.21	-0.18	0.62	14.78	-13.29	-0.92
4 G-C	-0.27	0.00	-0.40	5.39	-6.68	2.17
5 C-G	-0.23	0.05	0.08	6.00	-10.06	-0.24
6 C-G	0.48	-0.35	0.62	-8.82	-14.44	-0.69
7 G-C	0.46	-0.18	0.08	1.70	6.92	-2.16
8 C-G	-0.22	-0.21	0.02	6.36	-19.35	-1.80
9 G-C	-0.24	-0.26	-0.40	1.61	-18.84	3.63
10 C-G	0.19	-0.30	0.57	-9.46	-11.89	-2.23
11 G-C	0.30	-0.14	0.56	-2.12	-12.63	-3.86
12 G-C	-0.40	-0.36	0.17	4.04	-4.32	-0.29
13 C-G	-0.11	-0.06	-1.02	25.85	-5.88	2.36
14 C-G	0.19	-0.21	-0.40	-6.61	8.05	-3.99
15 G-C	-0.37	-0.11	-0.30	-13.58	-19.79	0.98
16 C-G	0.50	-0.18	-0.03	-17.61	-4.04	-0.05

step	Shift	Slide	Rise	Tilt	Roll	Twist
1 GC/GC	0.57	0.30	2.89	-3.93	4.48	32.84
2 CG/CG	-0.17	0.80	3.69	1.37	8.40	30.17
3 GG/CC	-0.65	-0.75	3.56	5.94	10.16	29.32
4 GC/GC	0.98	-0.05	3.38	-0.02	3.56	30.86
5 CC/GG	0.37	-0.88	3.74	-0.34	-0.67	44.07
6 CG/CG	0.34	0.75	3.15	4.61	-2.17	38.61
7 GC/GC	-1.01	0.18	3.45	-1.03	-10.44	31.04
8 CG/CG	----	----	----	----	----	----
9 GC/GC	0.73	0.45	3.74	-5.15	-3.73	36.70
10 CG/CG	-1.08	0.87	3.14	-2.11	3.16	37.98
11 GG/CC	0.50	-1.22	3.45	3.48	-2.12	28.71
12 GC/GC	0.16	-0.56	3.01	12.14	7.33	36.20
13 CC/GG	-0.38	-2.04	4.37	-8.48	10.57	30.39
14 CG/CG	-0.48	-1.19	4.04	3.89	-2.38	33.38
15 GC/GC	-0.54	0.18	3.53	-3.19	4.47	44.83

step	X-disp	Y-disp	h-Rise	Incl.	Tip	h-Twist
1 GC/GC	-0.14	-1.56	2.82	7.84	6.87	33.36
2 CG/CG	-0.35	0.61	3.76	15.76	-2.58	31.32
3 GG/CC	-3.34	2.34	2.96	19.13	-11.18	31.55
4 GC/GC	-0.80	-1.84	3.36	6.66	0.05	31.06
5 CC/GG	-1.10	-0.53	3.75	-0.89	0.46	44.08
6 CG/CG	1.38	0.03	3.12	-3.27	-6.94	38.94
7 GC/GC	2.21	1.61	3.25	-18.84	1.86	32.73
8 CG/CG	----	----	----	----	----	----
9 GC/GC	1.26	-1.91	3.55	-5.87	8.10	37.23
10 CG/CG	0.94	1.39	3.25	4.84	3.23	38.16
11 GG/CC	-1.93	-0.17	3.56	-4.24	-6.97	28.99
12 GC/GC	-1.67	1.12	2.76	11.28	-18.68	38.79
13 CC/GG	-5.80	-1.12	3.47	19.02	15.26	33.21
14 CG/CG	-1.57	1.61	4.04	-4.12	-6.73	33.68
15 GC/GC	-0.21	0.39	3.56	5.84	4.16	45.14

S6 d. Table: Local base-pair, local base-pair step and local base-pair helical parameters of the MD simulated structure at 310K and pH =8

bp	Shear	Stretch	Stagger	Buckle	Propeller	Opening
1 G-C	-0.31	-0.30	0.44	2.52	-6.65	-1.78
2 C-G	0.76	-0.03	0.32	-10.41	-15.05	9.43
3 G-C	-0.25	-0.31	0.13	-2.30	-8.48	-2.62
4 G-C	-0.39	-0.32	-0.46	-13.63	-36.90	2.14
5 C-G	0.39	0.08	0.33	-25.77	-15.06	4.45
6 C-G	-0.36	-0.10	-0.65	0.09	1.64	-0.25
7 G-C	0.44	-0.07	0.98	11.46	-20.55	-0.97
8 C-G	0.31	-0.25	-1.17	30.07	-14.95	0.82
9 G-C	-0.38	-0.33	0.73	-2.72	-24.11	-2.41
10 C-G	0.41	-0.29	0.29	-9.76	-9.63	-2.39
11 G-C	-0.59	-0.03	-0.10	-22.06	-9.65	0.83
12 G-C	-0.07	-0.15	-0.22	-10.55	-3.60	-7.22
13 C-G	-0.09	-0.04	-0.32	0.14	2.42	1.80
14 C-G	-0.15	-0.08	-0.58	22.80	1.77	0.08
15 G-C	-0.35	-0.35	0.34	-6.90	-34.03	-5.87
16 C-G	0.24	-0.13	-0.32	7.05	4.32	0.87

step	Shift	Slide	Rise	Tilt	Roll	Twist
1 GC/GC	0.37	0.02	3.81	2.72	4.98	28.65
2 CG/CG	-0.42	2.51	3.41	1.30	10.31	21.53
3 GG/CC	0.19	2.31	3.92	-0.73	10.34	42.40
4 GC/GC	1.12	-0.66	3.78	-1.79	-0.16	42.24
5 CC/GG	-0.09	-0.15	2.97	6.40	3.51	28.18
6 CG/CG	0.63	-0.99	2.97	-12.89	12.51	21.81
7 GC/GC	-0.66	0.58	3.09	17.18	-12.24	27.12
8 CG/CG	----	----	----	----	----	----
9 GC/GC	-0.11	-0.94	3.50	5.45	-3.65	44.52
10 CG/CG	-0.31	1.67	3.85	6.70	-1.75	44.40
11 GG/CC	0.59	1.42	3.14	-4.19	12.45	31.68
12 GC/GC	0.61	-0.15	3.11	2.09	2.84	22.45
13 CC/GG	0.53	2.43	3.12	10.69	10.55	37.83
14 CG/CG	-0.55	1.70	4.46	-14.97	9.94	36.89
15 GC/GC	0.21	-0.36	3.07	3.29	5.67	25.91

step	X-disp	Y-disp	h-Rise	Incl.	Tip	h-Twist
1 GC/GC	-1.23	-0.04	3.77	9.94	-5.43	29.19
2 CG/CG	1.98	1.52	4.13	25.76	-3.26	23.88
3 GG/CC	1.84	-0.35	4.34	14.05	0.99	43.59
4 GC/GC	-0.89	-1.76	3.73	-0.22	2.48	42.28
5 CC/GG	-1.00	1.45	2.84	7.06	-12.87	29.09
6 CG/CG	-4.35	-3.68	1.58	27.76	28.62	28.18
7 GC/GC	2.64	3.46	1.93	-22.32	-31.31	34.24
8 CG/CG	----	----	----	----	----	----
9 GC/GC	-0.87	0.67	3.52	-4.79	-7.15	44.98
10 CG/CG	2.37	1.11	3.70	-2.30	-8.80	44.91
11 GG/CC	0.39	-1.68	3.34	21.67	7.29	34.23
12 GC/GC	-1.35	-0.83	3.11	7.23	-5.34	22.72
13 CC/GG	2.23	0.52	3.66	15.55	-15.75	40.60
14 CG/CG	0.83	-1.63	4.63	14.69	22.12	40.89
15 GC/GC	-2.16	0.34	2.92	12.39	-7.19	26.72

S6 e. Table: Local base-pair, local base-pair step and local base-pair helical parameters of the MD simulated structure at 310K and pH =9

bp	Shear	Stretch	Stagger	Buckle	Propeller	Opening
1 G-C	-0.09	-0.09	0.65	-3.87	-21.71	-1.86
2 C-G	0.14	-0.27	0.63	-6.97	8.03	-1.10
3 G-C	0.15	-0.26	-0.21	-21.50	-31.43	0.41
4 G-C	0.03	-0.26	0.35	-19.35	-7.49	-3.03
5 C-G	0.15	-0.14	0.28	0.28	-14.62	-2.57
6 C-G	0.27	-0.08	-0.27	3.27	-25.56	5.80
7 G-C	-0.70	0.55	0.55	-23.29	-10.54	11.74
8 C-G	0.48	-0.11	0.34	-10.81	12.10	7.42
9 G-C	-0.45	-0.24	-0.48	-17.26	10.37	-2.36
10 C-G	0.40	-0.03	-0.05	12.47	-3.24	3.69
11 G-C	-0.02	-0.24	-0.22	-15.34	-14.58	-0.17
12 G-C	-0.38	-0.31	0.18	15.54	-2.53	-2.70
13 C-G	0.39	0.12	0.04	13.19	-2.77	6.45
14 C-G	0.12	-0.11	-0.45	1.95	-14.13	-0.44
15 G-C	-0.25	-0.18	-0.06	-1.36	0.20	-0.93
16 C-G	0.26	-0.25	-0.34	4.24	-18.99	1.57

step	Shift	Slide	Rise	Tilt	Roll	Twist
1 GC/GC	0.32	0.05	3.63	0.44	-8.03	37.05
2 CG/CG	-1.32	1.26	3.84	3.63	2.92	37.32
3 GG/CC	-0.05	-0.72	3.26	-8.39	4.69	35.59
4 GC/GC	0.42	-0.41	3.25	0.76	7.51	26.80
5 CC/GG	0.66	-1.03	3.04	7.36	9.21	34.87
6 CG/CG	1.07	0.43	3.95	-1.51	3.94	37.92
7 GC/GC	-0.21	0.36	3.23	-0.51	3.10	34.28
8 CG/CG	----	----	----	----	----	----
9 GC/GC	-0.52	0.80	2.91	-3.49	-0.86	31.85
10 CG/CG	-0.21	-2.03	4.00	2.50	8.02	25.69
11 GG/CC	-0.72	0.76	2.87	-1.61	2.81	38.14
12 GC/GC	0.29	0.57	3.47	1.61	0.02	30.33
13 CC/GG	0.47	-1.06	3.60	6.15	-6.83	38.72
14 CG/CG	-1.20	-0.49	3.63	-3.32	10.37	27.71
15 GC/GC	-0.59	0.02	3.31	4.77	2.44	34.32

step	X-disp	Y-disp	h-Rise	Incl.	Tip	h-Twist
1 GC/GC	1.21	-0.43	3.55	-12.45	-0.69	37.88
2 CG/CG	1.51	2.60	3.78	4.54	-5.65	37.60
3 GG/CC	-1.77	-1.04	3.08	7.50	13.43	36.82
4 GC/GC	-2.62	-0.69	3.04	15.81	-1.60	27.82
5 CC/GG	-2.78	-0.12	2.77	14.85	-11.87	36.75
6 CG/CG	0.06	-1.87	3.93	6.04	2.32	38.14
7 GC/GC	0.13	0.28	3.25	5.25	0.87	34.42
8 CG/CG	----	----	----	----	----	----
9 GC/GC	1.60	0.37	2.93	-1.57	6.33	32.04
10 CG/CG	-6.65	1.17	3.19	17.46	-5.44	27.01
11 GG/CC	0.84	0.91	2.94	4.29	2.45	38.27
12 GC/GC	1.08	-0.22	3.48	0.04	-3.07	30.38
13 CC/GG	-0.65	0.13	3.75	-10.13	-9.12	39.75
14 CG/CG	-3.35	1.57	3.35	20.67	6.61	29.74
15 GC/GC	-0.34	1.72	3.20	4.10	-8.03	34.73
