# Supplementary material for: Understanding the self-assembly dynamics of A/T absent ‘four-way DNA junctions with sticky ends’ at altered physiological conditions through molecular dynamics simulations
Source: PLoS One. 2023 Feb 8;18(2):e0278755. doi: 10.1371/journal.pone.0278755 (PMC9907842; doi:10.1371/journal.pone.0278755)
Supplement: Attachment — Submitted filename: Response to Reviewers_2.docx [file pone.0278755.s008.docx]

**#Journal Requirements:**

Please review your reference list to ensure that it is complete and correct. If you have cited papers that have been retracted, please include the rationale for doing so in the manuscript text, or remove these references and replace them with relevant current references. Any changes to the reference list should be mentioned in the rebuttal letter that accompanies your revised manuscript. If you need to cite a retracted article, indicate the article’s retracted status in the References list and also include a citation and full reference for the retraction notice.

**Response**: Thank you very much for the suggestion. The reference list have been reviewed and corrected. As per suggestion, some references have been replaced by other relevant current references. Changes have been highlighted in the revised manuscript with track changes. Retracted references have not been included in the manuscript.

**# Review Comments to the Author**

**Reviewer** #1: The Authors addressed all of my comments in detail. I am OK with these explanations and changes made to manuscript. It can be now published in PLOS ONE.

**Response**: Thanks to the Reviewer for his commend.

**Reviewer** #2: The manuscript in its revised version has improved massively as in my view all concerns raised by reviewers were addressed. From that point of view the manuscript may be accepted for publication.

**Response**: Thanks to the Reviewer for his commend.

**Comment**: However, I found some issues with the captions of Figures 5, 13 and 14 as the description of some of the images is missing or misleading. Authors must revise this.

**Response**: Thank you for pointing out. It is typing mistake, which have now been corrected in the re-revised manuscript.
